# Chalcogen Impurity Barriers in 2D Systems via Semi-Empirical/Machine Learning Modeling: A Survey of 4000 Materials

**DOI:** 10.1021/acs.jctc.6c00350

**Published:** 2026-07-20

**Authors:** M. L. Pereira, M. G. E. da Luz, P. Cesana, A. L. da Rosa, M. J. Piotrowski, D. Guedes-Sobrinho, T. A. S. Pereira, E. A. Moujaes, A. C. Dias, R. M. Tromer

**Affiliations:** † College of Technology, Department of Electrical Engineering, University of Brasília, Distrito Federal, Brasília 70910-900, Brazil; ‡ Department of Materials Science and NanoEngineering, Rice University, Houston, Texas 77005, United States; § Departamento de Física & Multidisciplinary Laboratory for Modeling and Analysis of Data in Complex Systems (MADComplex), Núcleo de Modelagem e Computação Científica-Centro Interdisciplinar de Ciência, Tecnologia e Inovação, Universidade Federal do Paraná, 81531-980 Curitiba, Paraná, Brazil; ∥ Institute of Mathematics for Industry, 12923 Kyushu University, 744 Motooka, Fukuoka 819-0395, Japan; ⊥ Institute of Physics, Federal University of Goiás, Campus Samambaia, 74690-900 Goiânia, Goiás, Brazil; # Department of Physics, Federal University of Pelotas, P.O. Box 354, Pelotas, Rio Grande do Sul 96010-900, Brazil; ¶ Chemistry Department, Federal University of Paraná, CEP 81531—980, 81531-980 Curitiba, Brazil; ∇ Physics Graduate Program, Institute of Physics, Federal University of Mato Grosso, 78060-900 Cuiabá, Mato Grosso, Brazil; ○ Instituto de Física, Universidade Federal Fluminense, Niterói, Rio de Janeiro 24210-346, Brasil; ⧫ National Institute of Science and Technology on Materials Informatics, Campinas 13087-548, Brazil; †† Institute of Physics, Federal University of Bahia, Campus Ondina, 40170-115 Salvador, Bahia, Brazil; ‡‡ Institute of Physics and International Center of Physics, University of Brasília, Distrito Federal, 70919-970 Brasília, Brazil; §§ Institute of Physics, University of Brasília, Distrito Federal, Brasília 70910-900, Brazil

## Abstract

Adequate characterization of two-dimensional (2D) materials with low energy barriers for impurity adsorption is key for advancing applications based on catalysis, sensing, and surface functionalization. However, first-principles methods, such as density functional theory, are often computationally extremely expensive for feasible large-scale screenings. Given such a scenario, we address a data-driven approach, which integrates the semiempirical extended Hückel method (EHM) with machine learning (ML) techniques to estimate adsorption energy barriers in the case of three relevant chalcogen impurities, sulfur (S), selenium (Se), and tellurium (Te). With this aim, we consider the 4036 2D materials found in the Computational 2D Materials Database (C2DB). The scheme employs the EHM to compute energy profiles along three in-plane migration paths, from which average barriers can be derived. The equilibrium distance between the impurity and the 2D surface is not calculated from a time-consuming geometry optimization. Instead, it is estimated from a simple effective phenomenological expression. Physicochemical descriptors are then obtained from the Matminer (Materials Data Mining) library for curated features. Four different ML models are tested, with XGBoost (considering hyperparameter optimization via Optuna) leading to the highest performance. We further use SHAP to verify the resulting predictions, focusing on the ∼1500 materials displaying the lowest barrier values. As could be anticipated, we establish that the average valence electron count, electronegativity, and atomic number are typically the most relevant attributes to validate the ML model. However, we are also able to determine, for the different chalcogen atoms, which other few descriptors likewise considerably influence the adsorption properties. Our results show that when combined with interpretable ML protocols, EHM (and potentially semiempirical calculations in general) can produce a scalable framework for choosing 2D structures that exhibit the desired capture/release dynamics pertinent in a variety of utilization.

## Graphical abstract

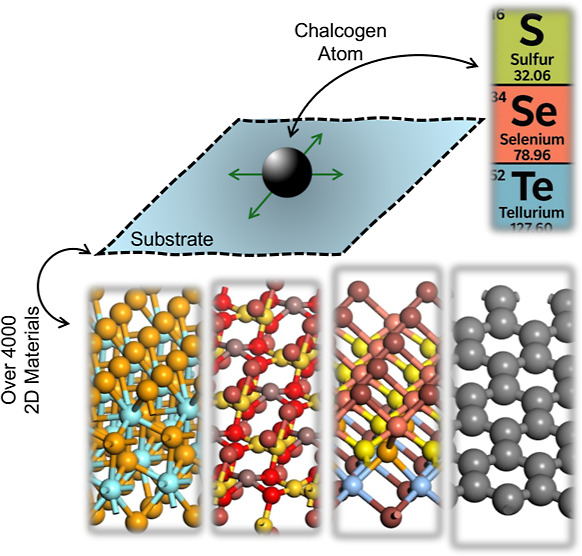

## 1 Introduction

Two-dimensional (2D) materials have emerged as one of the most promising classes of systems for advanced technological applications, owing to their unique physical and chemical properties. For example, transition metal dichalcogenides (TMDs)—such as molybdenum disulfide (MoS_2_), diselenide (MoSe_2_), and ditelluride (MoTe_2_)—exhibit outstanding electronic, optical, mechanical, and catalytic characteristics.^1–4^ The versatility of these 2D layered structures positions TMDs as attractive candidates for various usages, including flexible electronics, high-performance sensors, optoelectronic devices, photocatalysis, and emerging energy storage technologies.

More broadly, some chalcogens have found a huge number of concrete applications.^5,6^ The full family corresponds to the six elements (O, S, Se, Te, Po, and Lv) of periodic table group 16. They are generally highly reactive, although their reactivity steadily decreases down the group. Oxygen exhibits a distinct character within the chalcogen family due to properties that differ significantly from those of its heavier congeners. In contrast, polonium and livermorium are radioactive, with the latter being a synthetic element. On the other hand, the nonmetals sulfur (S) and selenium (Se) and the metalloid tellurium (Te) share many similar characteristics.

The technological relevance of the above-mentioned last three elements, associated with 2D structures, is demonstrated across several domains; for instance, S and Se are utilized to improve the efficiency of large-scale 2D film exfoliation,^7^ while Se and Te atoms embedded in molecular motifs drive chiral on-surface molecular recognition via chalcogen bonding.^8^ Furthermore, Te adsorption enables the fabrication of extended 2D supramolecular network architectures on metal surfaces without altering the molecules’ electronic properties.^9^ In the field of energy and devices, substituting surface atoms with chalcogen heteroatoms activates the basal plane of 2D layers for high-density single-atom catalysis,^10^ and incorporating S or Se into Te-based alloys significantly optimizes the sensitivity and signal-to-noise ratio in room-temperature thin-film gas sensors.^11^

Among the distinct properties of 2D structures, the energy barrier arising from the interaction with atoms or molecules is particularly crucial. Indeed, this plays a central role in several physicochemical processes, such as molecular adsorption,^12^ charge transfer,^13^ surface catalysis,^14^ and chemical sensing.^15^ Hence, when focusing on particular utilization, an assessment of appropriate structures with low energy barrier values across a wide range of TMDs may be essential.^16^

Density functional theory (DFT) stands out as one of the primary calculation tools for accurately obtaining nanomaterials’ structural and electronic properties. However, DFT’s high computational cost significantly limits its systematic application to the evaluation of energy barriers over big material data sets.^17,18^ This limitation poses a significant obstacle to a large-scale exploration of promising systems, such as those in the Computational 2D Materials Database (C2DB). Actually, the C2DB comprises thousands of 2D compounds, most of them still largely unexplored.^19^

To overcome these computational bottlenecks, semiempirical approaches offer a viable alternative. In this direction, the extended Hückel method (EHM) provides a computationally inexpensive framework that balances speed with qualitative accuracy, rendering it ideal for large-scale electronic and energy estimations.^20,21^ While EHM obviously lacks the precision of DFT due to the absence of a self-consistent field loop, explicit electron–electron interactions, and reliable geometry optimization capabilities, it is highly useful for screening total energies across vast pools of nanostructures.^22^ It effectively acts as a primary selective filter to isolate top candidates within practical time constraints. For instance, this screening strategy has successfully predicted the adsorption energies of contaminants on 2D surfaces,^23–27^ as well as the behavior of alkali metals (Na, K, and Li) on graphene.^28–30^

In parallel, machine learning (ML) techniques have revolutionized the study of material features, enabling the efficient prediction of complex physical quantities from a limited number of reference calculations.^31^ Moreover, the application of ML in high-throughput probing should facilitate trend identification and accelerate the uncovering of materials with tailored properties. Certainly, a critical step in the scheme is the construction of representative descriptors for systems under scrutiny. However, a robust tool to address such difficulty is the Matminer (Materials Data Mining) library, skillfully generating effective quantitative representations for ML models.^32^

Among the many available supervised learning algorithms, XGBoost (Extreme Gradient Boosting) has emerged as a high-performance protocol for handling heterogeneous elaborated data sets.^33,34^ Further, to enhance model interpretability, techniques such as SHAP (SHapley Additive exPlanations) have been increasingly considered, allowing for the quantification of descriptor importance and for the inference of actual physical correlations. This establishes explicit connections between the most relevant features and the underlying physicochemical mechanisms governing the system-predicted properties.^35,36^

Here, we develop a novel framework to investigate energy barriers in 2D structures by integrating EHM with advanced ML techniques. We then address the chalcogen atoms S, Se, and Te, interacting with 2D material surfaces. We take into account the problem geometry through a phenomenological, nevertheless rather simple and effective, expression based on the covalent radii of the adsorbates. It gives the fixed equilibrium distance between the atom and the 2D surface. From the proposed methodology, we estimate the energy barriers for over 4000 systems retrieved from the C2DB database. The obtained large data set is thus used to train predictive models (in different ranges of energy) employing XGBoost—for the sake of comparison, other ML procedures are also tested. Finally, analyses considering the SHAP protocol for the 1495 materials with the lowest barriers lead to accurate predictions and tangible physical interpretation of the factors governing energy barriers in these structures. The generality of the whole procedure might constitute a relevant new approach for studying distinct aspects of atoms deposition on 2D materials.

## 2 Methodology

In the present study, we follow a sequential protocol that involves (see the workflow in Figure 1): (i) to build an energy barrier data set (steps 01 and 02); (ii) to extract physicochemical descriptors for numerical representation of each 2D system (step 03); and (iii) to implement ML models based on the physicochemical descriptors (step 04) and then to physically interpret the obtained results (step 05).

**Figure 1:**
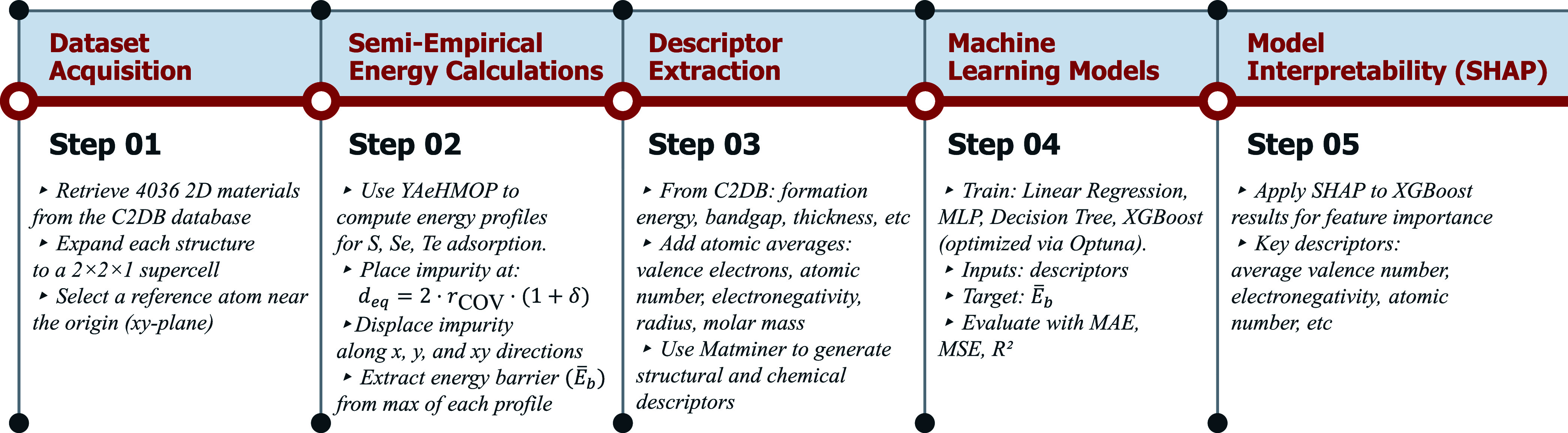
Workflow summarizing all the data gathering, calculations, ML models, and interpretative analyses implemented to properly characterize the adsorption of chalcogen atoms in 2D materials classified in the C2DB database (comprising 4000+ distinct systems).

For the actual systems, we accessed one of the most comprehensive repositories of 2D structures, the C2DB,^19^ covering a rich diversity of configurations, symmetries, and fundamental physicochemical properties. It comprises an extensive collection of 2D materials, from experimentally synthesized ones, such as graphene, to compounds proposed either theoretically or identified through AI-assisted simulations.

Our framework steps are described below.

### 2.1 Semiempirical Calculations

For each structure available in the C2DB, we estimated the energy barrier associated with the adsorption of three types of dichalcogenide impurities, sulfur (S), selenium (Se), and tellurium (Te), by employing the EHM, as in the YAeHMOP software package.^37^ This semiempirical method can calculate the system total energy, but without performing any geometry optimization, which requires a predefined equilibrium distance *d*_eq_ between the impurity and the 2D surface; refer to Figure 2. This *d*_eq_ is thus estimated from the scheme proposed in Section 2.1.1.

**Figure 2:**
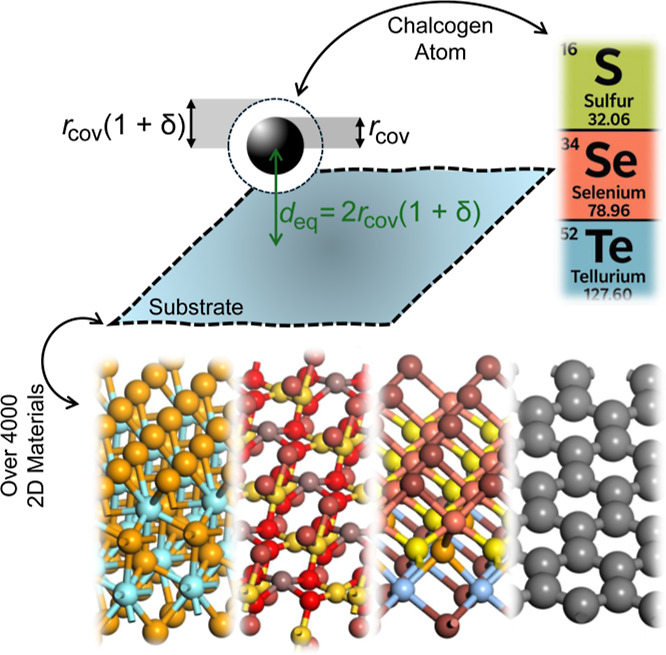
Schematics of the problem geometry. Distinct 2D materials (from the C2DB database) constituting the substrate for a chalcogen atom (either S, Se, or Te) adsorption. The effective equilibrium distance, *d*_eq_, used to calculate the energy barriers, is estimated from the phenomenological considerations in Section 2.1.1.

Before detailing the concrete steps of our methodology, a brief discussion regarding the reliability and transferability of the EHM across chemically diverse 2D materials is in order. First, we recall that evaluating reaction pathways and energy barriers across thousands of compounds remains computationally impractical using standard first-principles methods.^38,39^ To bypass this limitation, semiempirical approaches like the EHM provide a viable alternative.^40^ Naturally, the EHM presents inherent limitations regarding transferability. Because its parameters are derived from atomic or small-molecule reference data—and lack the self-consistent treatment of ab initio approaches discussed in the Introduction—the method’s quantitative accuracy expectedly can vary considerably among chemically distinct classes of 2D materials within the C2DB database. This is potentially the case in environments featuring highly ionic bonds, large electronegativity differences, strong electron correlations, or complex transition-metal d-orbital physics.^40^ Nevertheless, when calibrated against first-principles data, optimized EHM parameters retain sufficient transferability to reliably reproduce qualitative energetic orderings and band structure topologies in structurally related phases; see, for instance, the procedures proposed in refs 40–42 . Targeting over 4000 distinct 2D materials, this work does not aim for absolute quantitative predictions; instead, the EHM serves as a preliminary screening tool to map qualitative trends and classify adsorption energy barriers.^39^ By coupling this fast framework with interpretable ML, we aim to partly mitigate transferability limitations, establishing a preliminary filter to narrow down the vast chemical space.^39,43^ This screening workflow allows computationally intensive ab initio procedures or targeted experiments to be deployed later in a more focused manner.^39,43^

Each 2D material was expanded into a 2 × 2 × 1 supercell, and a reference atom was selected as the one closest to the origin within the *xy* plane. The impurity was initially placed above this reference atom at the equilibrium distance *d*_eq_. Subsequently, the impurity was displaced along three distinct in-plane trajectories: the *x*-axis, the *y*-axis, and the *xy* diagonal. Each trajectory spanned a displacement of approximately 3 Å, beyond which edge effects—due to the cluster approximation adopted in YAeHMOP for periodic boundary conditions—began to introduce nonphysical artifacts in the energy profile. Therefore, three energy barrier curves were generated for each system, corresponding to the three directions. The average energy barrier was computed as

1 $${\mathrm{E̅}}_{b}=\frac{1}{3}\left(E_{b}^{x}+E_{b}^{y}+E_{b}^{xy}\right)$$

where the maximum value from each energy profile was selected as the target variable for ML models.

#### 2.1.1 The Semiempirical Equilibrium Distance

2D materials are often characterized by in-plane covalent bonds and out-of-plane van der Waals interactions.^44^ The latter is responsible, for example, for the separation distance between graphene sheets in distinct bilayer structures (typically in the range 3.25–3.50 Å)^45^ and for atom adsorption.^46,47^ Thus, the van der Waals radius^48^—associated with an effective size for the interfacial adhesion mechanism—is one of the main factors determining the equilibrium distance *h*_eq_ between the adsorbed atom and distinct points along the 2D material surface.^49^ Moreover, some findings in the literature^50,51^ have pointed out that atom adsorption in 2D materials tends to display rather simple universal relations to the adsorbent structure geometrical symmetries. Hence, in a first order approximation, where we suppose an effective equilibrium distance *d*_eq_ for the full surface, such a configuration parameter can be assumed fairly independent of specific details of the atoms and 2D materials involved (but see additional discussion in Section 4).

Based on the above, we propose to set *d*_eq_ = 2*r*_vdW_, with *r*_vdW_ being the van der Waals radius. Nonetheless, it has long been known^48,52^ that for a large number of situations *r*_vdW_ – *r*_cov_ = Δ_*r*_ > 0, where *r*_cov_ is the atom covalent radius and Δ_*r*_ is reasonably constant. So, *d*_eq_ can be written as (see the schematics in Figure 2)­

2 $$d_{eq}=2r_{cov}\left(1+\delta \right)$$

where δ = Δ_*r*_/*r*_cov_.

For weak van der Waals bonding (like in gases, liquids, and polymers), the Pauling rule proposes that Δ_*r*_|_Pauling_ = 0.8 Å, a value put on solid ground in ref 52. For the clearly tighter attraction between adsorbed atoms and 2D materials,^46,47^ such Δ_*r*_ must be smaller. So, based on some estimations in the literature (see, e.g., scheme 5 and Sec. 5.4 of ref 53), we assume Δ_*r*_ ∼ Δ_*r*_|_Pauling_/2 = 0.4 Å. Given that the three atoms of interest here are S, Se, and Te of *r*_cov_, equal to 1.02 Å, 1.2 Å, and 1.36 Å, respectively, we get δ ≈ 0.3. So, eq 2 with δ = 0.3 is the expression we consider throughout this work. As further support for the approximation in eq 2, we mention that it properly reproduces average *h*_eq_’s from DFT calculations for alkali metal atoms (Na, K, and Li) interacting with graphene and other 2D surfaces.^28–30^

Moreover, we have performed some simple numerical tests to estimate the impact of varying *d*_eq_ via the parameter δ. Minor fluctuations within ±10% do not qualitatively alter the calculated energetic trends or the materials’ ranking order, with Se and Te tracking fairly close and S exhibiting the lowest sensitivity. Conversely, a systematic overestimation of *d*_eq_ by 30% or more artificially flattens the potential energy profiles, triggering a drastic drop in the diffusion barriers across all host structures. Within the EHM framework, such extreme separations severely diminish the atomic orbital overlap, leading to an asymptotic decoupling between the adatom and the 2D surface that erases the distinct electronic features of the substrates and drives the barriers toward a uniform, artificially low regime.

As a final test to benchmark the fixed effective distance approximation, DFT calculations were performed using graphene as a reference substrate. Substituting a dynamically fluctuating migration height with a fixed distance introduces expected quantitative trade-offs. DFT optimizations reveal that the impurity height fluctuates along the diffusion pathway, ranging from 1.65 Å to 2.18 Å (average 1.78 Å) for S, 2.28 Å to 3.36 Å (average 2.94 Å) for Se, and 1.97 Å to 2.15 Å (average 2.04 Å) for Te. The fixed model distances (2.65 Å for S, 3.12 Å for Se, and 3.54 Å for Te) overestimate these minimum binding heights by 60.6%, 36.8%, and 79.7%, respectively. Crucially, against path-averaged DFT heights, this overestimation drops to 48.9% for S and a remarkably close 6.1% for Se, demonstrating that a fixed distance physically captures the regular path-averaged environment rather than just the localized global minimum. Interestingly, these results point out that future phenomenological refinements should systematically decrease *d*_eq_, consequently moving it away from the unphysical behavior mentioned above at larger distances.

### 2.2 The Physicochemical Descriptors

To numerically characterize each system (see Figure 1, step 03), we considered a set of physicochemical descriptors derived directly from the C2DB database; as examples, we cite formation energy, monolayer thickness, and electronic band gap. These were complemented by externally computed attributes, including average valence electrons, atomic number, atomic radius, electronegativity, and molar mass. In addition, we employed the Matminer library,^32^ which provides a broad range of automated descriptors based on the chemical composition, crystal structure, and electronic features. Matminer has been widely adopted in materials informatics due to its ability to generate robust, interpretable, and reproducible properties for use in ML pipelines.^54^

We call the totality of these features, namely, those directly from the C2DB, plus the computed attributes, plus those from Matminer, the full set of descriptors, FSD.

### 2.3 The ML and Interpretability Models Considered

The generated and assembled data set of descriptors, the FSD, was considered to evaluate and compare the performance of four supervised learning algorithms. The first was a simple model that assumes a linear relationship between the descriptors and the target energy barrier. Although interpretable and straightforward, linear regression is naturally insufficient for systems with considerable nonlinear trends.^55^ Next, we employed a multilayer perceptron (MLP) neural network, capable of capturing complex polynomial patterns, nonetheless requiring a great amount of data and hyperparameter tuning.^56^ As a third possibility, we tested a decision tree model that works through hierarchical decision rules by partitioning the descriptor space. While highly interpretable, this approach is susceptible to overfitting of noisy data.^57^

Lastly, we assumed the XGBoostalgorithm, which is a powerful ensemble method based on gradient-optimized decision trees. XGBoost is greatly recognized for its highly predictive accuracy, resilience to overfitting, and excellent performance across large and heterogeneous data sets. Indeed, in our analyses, this scheme outperformed all the other three protocols.^33^

To interpret the outputs of the ML models and to identify the most influential features governing energy barrier predictions, we used the SHAP method.^35^ Based on cooperative game theory, SHAP assigns a Shapley value to each input feature, quantifying its marginal contribution to the model’s prediction. SHAP has lately earned considerable popularity in materials science. It allows for concrete interpretations of results obtained from complex black-box models. In other words, SHAP helps to link physical attributes to forecast behavior from AI-based methodologies. This is fundamental to ascribe phenomenological significance to context-free procedures, especially if one is trying to unveil the physical-chemical mechanisms underlying the properties of a material.^58^

## 3 Results

### 3.1 A Relevant Benchmark: Graphene as a Substrate

We start our analysis supposing graphene as the 2D substrate for the S, Se, and Te dichalcogenide elements, given the graphene’s well-known physical and chemical properties and its widespread usage as a reference in surface science. So, this is an ideal case (a benchmark) to validate the consistency and reliability of the proposed semiempirical methodology.

We present the energy barrier profiles obtained for S, Se, and Te, respectively, in Figure 3a–c. Each graph displays the variation in the energy barrier as a function of a generalized reaction coordinate, corresponding to three distinct in-plane diffusion paths for the impurity along the *x* (blue), *y* (orange), and diagonal *xy* (green) directions. These trajectories simulate the lateral migration of the adsorbate along the 2D surface, allowing us to characterize directional effects on diffusion. We recall that, as discussed in Section 2.1, the displacements were limited to approximately 3 Å, a constraint introduced to reduce edge effects from the simplified periodic boundary condition treated via cluster approximation by YAeHMOP software.

**Figure 3:**
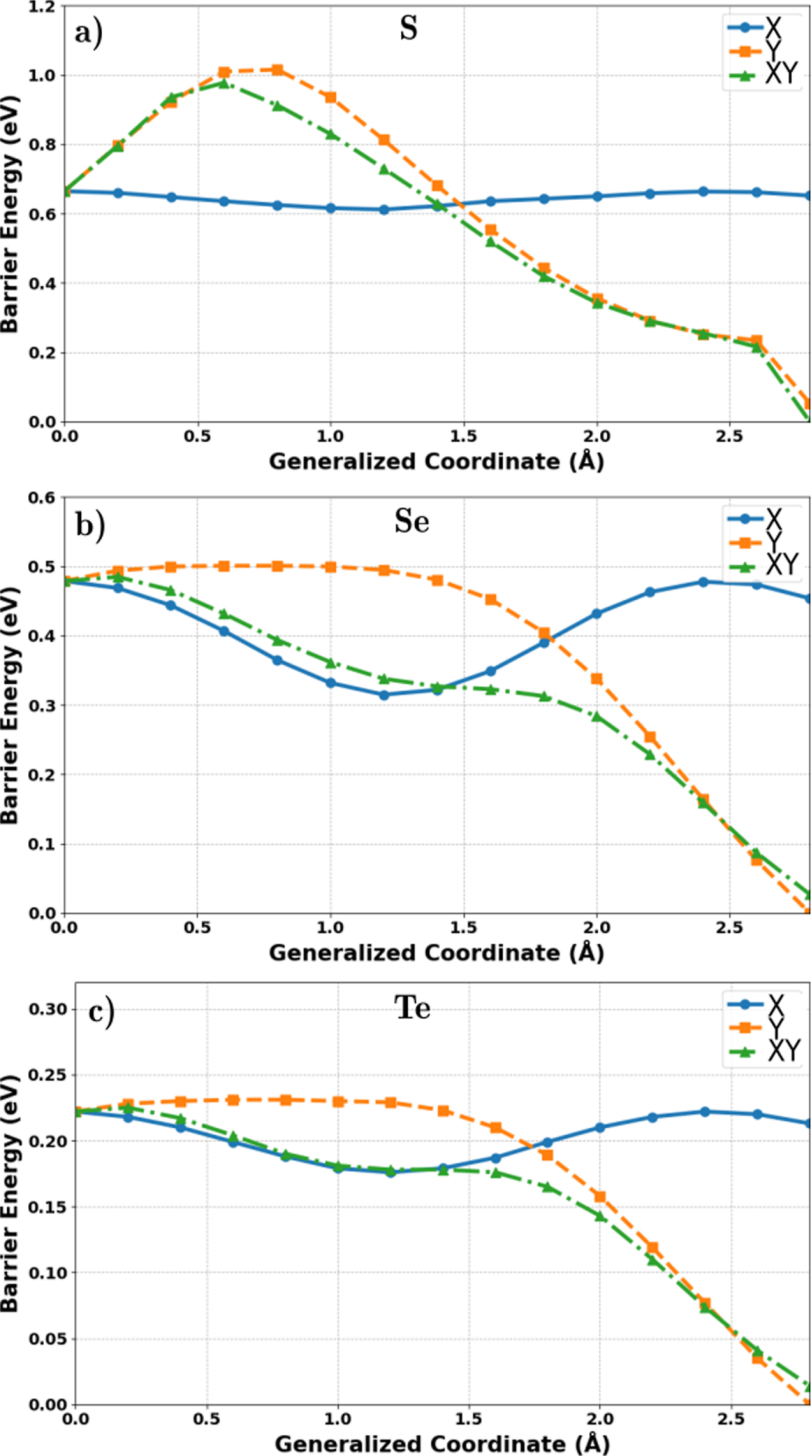
Energy barrier profiles (in eV) obtained for the dichalcogenide elements: (a) S, (b) Se, and (c) Te, adsorbed on graphene using the EHM via the YAeHMOP software. In each case, the impurity was displaced along three directions over the graphene surface: *x* (blue), *y* (orange), and the diagonal *xy* (green).

As clear from Figure 3a, the energy barrier profile for S shows marked anisotropy among the *x* and *y* displacement directions. Indeed, the global highest barrier takes place along the *y* direction, peaking at approximately 1.02 eV, whereas the highest barrier along the *x* direction reaches only 0.66 eV—but for a profile which is almost constant. Mostly influenced by *y* (in fact, closely following it), the *xy* path has a peak at 0.98 eV. This pronounced directional dependence seems to be attributed to the graphene surface’s local electronic topology and the S atom’s preferential adsorption orientation. Notably, anisotropic interactions have been reported in DFT-based computations,^59,60^ arising from variations in the registry between the impurity and the graphene lattice. Energy barrier values for S on various surfaces typically range from 0.5 to 1.2 eV, depending on the adsorption site and surface coverage.^61^ Our much simpler semiempirical calculations fall within this expected range.

Results for Se are depicted in Figure 3b. Note that overall, it displays a shorter range of energy variation than S. The *y* direction continues to have the largest barrier, reaching a maximum of roughly 0.50 eV; the *x* and *xy* directions show maximums that are only somewhat lower than this. Also, different from S, for Se, the *xy* path is now a more balanced combination of the behaviors along *x* and *y*. This decrease in magnitude and anisotropy might be attributed to the larger atomic radius and lower electronegativity of Se relative to those of S, weakening the orbital overlap with the graphene surface. Thus, the potential energy landscape becomes less corrugated with shallower energy minima and maxima.

These trends are consistent with theoretical studies of Se adsorption in graphene and related 2D materials, where typical barriers of ∼0.6 eV are often obtained.^60^ This enhanced surface mobility of Se can have practical implications for surface functionalization, epitaxial growth, and thermally activated diffusion. Hence, further characterization through ML approaches may provide valuable insights into the design and selection of functional materials (see later).

The barrier energies for Te are displayed in Figure 3c, exhibiting the lowest values among the three elements, although they have general traits resembling those of Se. The maximum is approximately 0.23 eV for *y* and about 0.22 eV for the *x*- and *xy*-directions. The Te quasi-isotropic behavior (in terms of maximum barrier heights) reflects its larger polarizability and atomic radius, which lead to weaker and more delocalized interactions with the graphene surface. Consequently, the energy landscape becomes flatter and is less sensitive to the diffusion direction. All this aligns with previous investigations of Te adsorption on graphene, yielding barriers usually below 0.3 eV.^62^ Thus, the EHM properly captures the correct interaction energy relative magnitudes and key symmetry trends, critical features for the understanding of surface diffusion and transport phenomena.

An important qualitative point to be emphasized is that, despite the simplicity of the semiempirical scheme used, particularly its lack of geometry optimization and electronic structure resolution, the order of the calculated energy barriers (S > Se > Te) concurs with the expected chemical tendency across the periodic table group 16: the increase in atomic radius and the decrease in electronegativity as one moves down the chalcogen group. This results in progressively weaker effective chemical interactions between the impurity and substrate.

### 3.2 Energy Barrier Computations for the Full C2DB Database

Having illustrated the reliability of the present approach for the paradigmatic graphene case, next we apply our semiempirical method to a rather large collection of systems. Indeed, for the 4036 2D materials from the C2DB database, each surface is doped with one of the three dichalcogenide impurities. Then, the average energy barrier is calculated from the maximum energy barrier along the *x*, *y*, and *xy* directions; refer to eq 1. In Figure 4, we summarize the simulations by means of histogram distributions (i.e., frequency or number of cases within a given energy interval, the bin). To simplify the analysis, we show only the materials with barrier energies of up to 5.0 eV. This reduces the number of materials to about 3150 (78% of the original set) for each impurity atom, namely, S (Figure 4a), Se (Figure 4b), and Te (Figure 4c).

**Figure 4:**
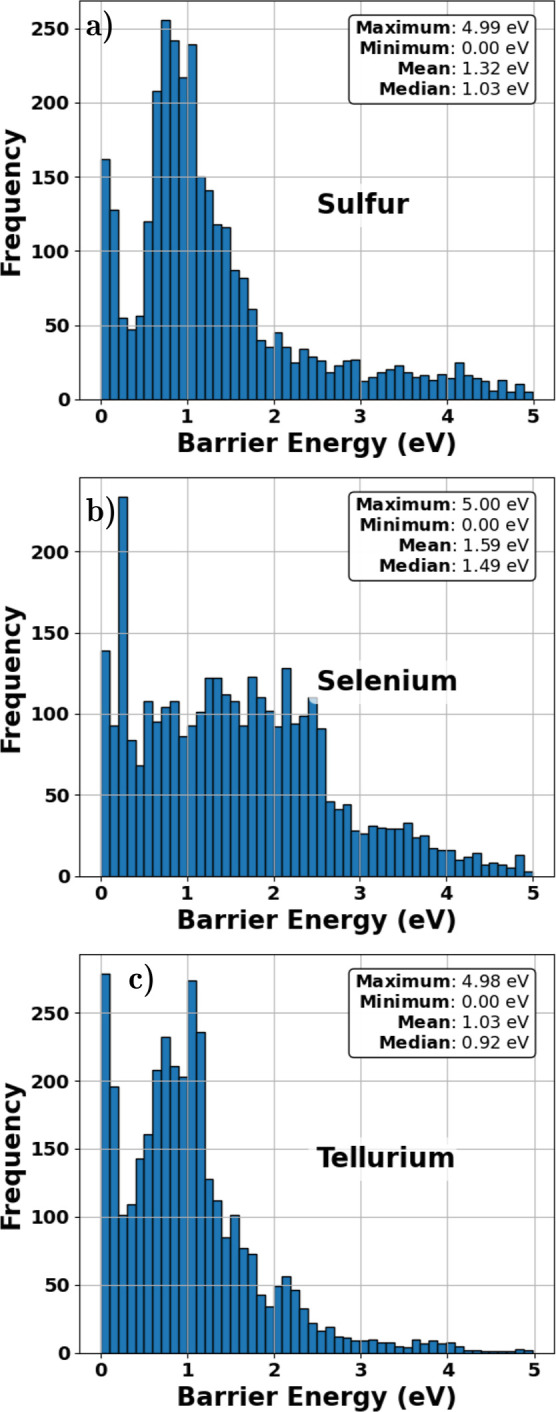
Histograms of average energy barriers for about 3150 2D systems from the C2DB database, for which the energies do not exceed 5.0 eV. The impurities are (a) S, (b) Se, and (c) Te. The values represent the average over the three directions *x*, *y*, and *xy*.

We should emphasize that the C2DB comprises both already synthesized as well as theoretically predicted 2D systems, and it covers many distinct structural families. In this way, the present survey characterizes the energy barrier behavior across a great variety of 2D materials. On the one hand, it pinpoints the variations in substrate interactions, influenced by structure, symmetry, and electronic properties. On the other hand, it unveils the differences in the chemical nature of the dichalcogenide adsorption. Therefore, the histogram reveals certain coincidences, as well as important distinctions between the three atoms.

As for similarities, for the energy interval considered, 0–5 eV, the majority of the examples are below 2.0 eV. Also, there is a considerable number of materials for which the barriers are in the range 0–0.3 eV. The mean and median for S, Se and Te are, respectively, 1.32 and 1.03 eV, 1.59 and 1.49 eV, and 1.03 and 0.92 eV. So, the difference between the highest (Se) and lowest (Te) mean, of about 35%, is not so large.

Regarding the contrasts among S, Se, and Te, we first observe that the histogram for S, as shown in Figure 4a, demonstrates a certain similarity among materials, with the principal mode being around 0.6–1.6 eV. Then, there is a long tail extending toward higher energies (up to 5 eV). Consequently, the distribution exhibits a considerable skewness. Further, it shows that although most systems present moderate barriers, a significant number of surfaces have a strong interaction with S. This is likely due to sulfur’s high propensity to form covalent bonds with the 2D materials’ orbitals of high electronic density. Such orbitals are particularly common in transition-metal-based structures.

In contrast, the Se histogram in Figure 4b displays a broad plateau, with fairly similar frequencies in the energy range 0.6–2.5 eV. The average for Se is 1.59 eV, while for S, it is 1.32 eV. Such a higher value can be attributed to a slightly lower electronegativity of Se, resulting in a more even interaction with the surfaces, but which is still enough to yield relevant barrier values. However, the tendency of a stronger orbital polarization for Se, which depends on the lattice symmetries—i.e., hexagonal, orthorhombic, etc.—may also contribute to a wider range for the energy barrier values.

Overall, the histograms for S and Te, shown in Figure 4a,c, respectively, display certain parallels; however, differences are also identified. For instance, the distribution for Te exhibits a faster decreasing tail. Moreover, the number of surfaces having barrier energies in the range 0–0.3 eV for S is only around 60% that for Te. These facts lead to a mean of 1.03 eV for Te (the lowest among the three impurities), suggesting that generally, Te interacts weakly with the 2D systems, the same trend found for graphene as a surface in the previous section. This should be expected, considering the Te larger atomic radius, lower electronegativity, and higher polarizability, when compared to S and Se. It is worth remarking that, from DFT-based computations, implemented for much fewer situations,^62^ the somewhat feeble binding nature of Te has been demonstrated. Here, from our relatively inexpensive protocol, we have been able to extend such predictions for a rather big set of 2D materials.

### 3.3 ML Model Predictions for the Barrier Energies

In critical applications, such as those related to catalysis and building functionalized surfaces with adjustable diffusion characteristics, adsorbed atoms should exhibit high surface mobility, which translates into low-energy barriers. Hence, for the ML analyses here, we further restrict the materials to those with energy barriers up to 2.0 eV. This amounts to around 2560 2D systems from the original 4036, i.e., 63% of the total number of materials in the C2DB database.

Although semiempirical calculations tend to display differences from the barriers’ actual values, the global statistical patterns observed in the distributions like spread, shape, mode position, and average energetic relative order (Se > S > Te) comply with known physicochemical traits and with much more sophisticated theoretical and experimental analyses in the literature. This supports the use of our obtained results as reliable input data^63^ to implement ML models. We also recall that the predictors considered for the ML models below (and for the SHAP study in the next section) are all those mentioned in Section 2, the FSD.

For the ML outcomes presented below, we stress that the parameters of each algorithm were thoroughly tested and tuned to optimize their predictive performance. More concretely, for simpler models, like linear regression and decision trees, various combinations of standard settings were explored, including maximum tree depth, splitting criteria, and normalization strategies. Further, an MLP architecture with various hidden layers was considered for the neural network, and several configurations entailing batch size, activation functions, and optimization schemes were tested. In the case of XGBoost, which delivered the best results, a fine-tuning procedure was carried out using the Optuna library.^64^ It allowed an efficient hyperparameter search, targeting the number of estimators, learning rate, maximum depth, and subsampling ratio (explicit values of parameters and further details are given in the Supporting Information). Such a protocol significantly improved the model accuracy—especially in the testing phase, where generalization capability is critical.

The comparison between the ML model performances yields essentially the same conclusions for the three adsorbing atoms addressed in this work. So, the following discussion focuses on the S case in detail, with only a brief summary provided for Se and Te. The scatter plots in Figure 5 for S compare the ML predictions (for all four models investigated) with the calculations of Section 3.2. In Figure 5, each row corresponds to one model, with the left (right) panels depicting the training (test) results. The performances are quantified through the mean absolute error (MAE), mean squared error (MSE), and the coefficient of determination (*R* ^2^), shown in the corresponding graphs.

**Figure 5:**
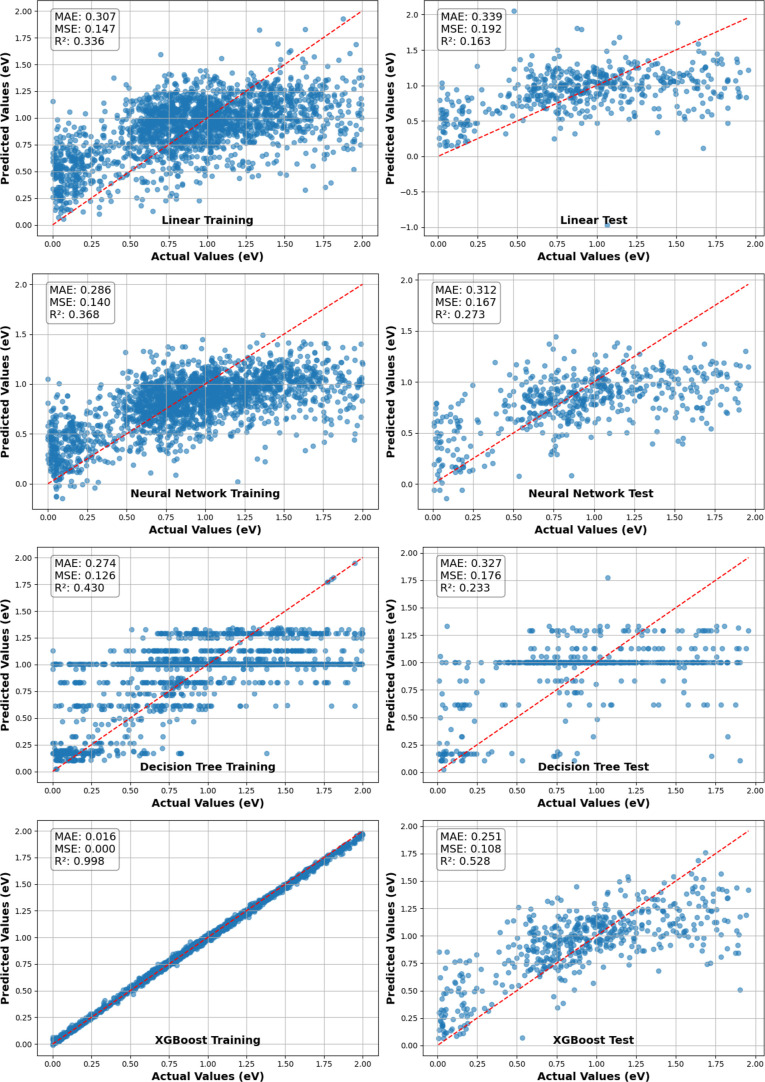
Comparison between the calculated and ML-predicted energy barrier values (≤2.0 eV) for the S impurity adsorbed on materials of the C2DB database. The ML models are Linear Regression (first row), Neural Network (second row), Decision Tree (third row), and XGBoost (fourth row). Each model training stage is shown on the left and the testing stage on the right. The dashed red line represents the ideal *y* = *x* identity line. Performance metrics, namely, MAE, MSE, and the coefficient of determination *R* ^2^, are also shown.

For the linear regression in the top row (certainly the simplest ML model), as expected, the performance is rather modest, with R^2^ equal to 0.336 and 0.163 for the training and testing sets, respectively. It also leads to an MAE close to 0.31 eV (training) and a very broad dispersion—refer to the diagonal dashed red line. This stems from a linear fitting trying to describe the nonlinear relationship between the assumed descriptors and the target energy barrier.

The second row in Figure 5 shows the results for the neural network model based on an MLP architecture. Now, *R* ^2^ and MAE are, respectively, 0.368 and 0.286 eV (training) and 0.273 and 0.312 eV (testing). Thus, some improvement is achieved, although it is still limited. This may be attributed to the relatively simple nature of the input features, based mainly on compositional averages, and data set size: neural networks are notoriously dependent on significantly large samples, despite being of low computational cost, particularly when coupled with architectural and hyperparameter optimization.^56^

The decision tree model is presented in the third row of Figure 5. It has an important feature of partitioning the input space through a hierarchy of decision rules. While leading to *R* ^2^ = 0.430 during the training stage, it suffered a notable drop in performance during testing, with *R* ^2^ decreasing to 0.233 and the MAE increasing from 0.274 to 0.327 eV. This behavior suggests overfitting, a well-documented limitation of single decision trees when applied to noisy or highly variable data.^65^ Also, the layer-like pattern in the plots reflects the discrete nature of the model predictions (owing to the countable rules), resulting in a poor representation of any target variable inherently continuous, of course, the case of energy barriers.

Finally, the fourth row of Figure 5 depicts the XGBoost outcomes, outperforming the other three models in every respect. During training, *R* ^2^ = 0.998, with negligible MAE and MSE, indicating an almost perfect fit. In the testing stage, XGBoost maintains robustness, with *R* ^2^ = 0.528, MAE = 0.251 eV, and MSE = 0.108. This success can be attributed to the model’s ensemble nature, combining many weak learners (shallow decision trees) through gradient boosting to minimize the prediction error. XGBoost also incorporates built-in regularization techniques, which mitigate overfitting. Such superiority has already been reported for predicting other material characteristics, e.g., band gaps, formation energies, elastic constants, and thermal coefficients.^66^

Akin analyses for Se and Te showed essentially the same qualitative results concerning the efficiency of the distinct ML models, again with XGBoost performing considerably better. For instance, for Se, the *R* ^2^ values in the test stage were 0.151, 0.309, 0.260, and 0.520 for linear regression, neural network, decision tree, and XGBoost, respectively. For Te, the corresponding values were 0.319, 0.353, 0.313, and 0.540. The full set of calculations for Se and Te are given in the Supporting Information.

The above upshots demonstrate that while simpler ML schemes, such as linear regression and decision trees, offer baseline insights, they lack the required generality and plasticity to unveil the intricate relationships between atomic descriptors and more tangled quantities, such as energy barriers. Even the more elaborate neural network approach yielded limited gains. On the contrary, XGBoost effectively displayed the proper balance between accuracy and stability to deal with a large and diverse set of materials.

### 3.4 Examining the ML Predictability

Considering the best-performing model, XGBoost, for the relatively broad barrier energy range of 0–2.0 eV, all chalcogen atoms exhibited strong consistency between cross-validation and held-out test performance. This indicates that the adopted hyperparameter optimization and data partitioning strategy effectively mitigated information leakage. However, the train–test error gap remained substantial (∼0.25–0.32 in RMSE), a clear sign of overfitting when the task covers a wide energy range with higher intrinsic variance.

Repeating the XGBoost modeling, but restricting the set of materials to those with barrier energies ≤1.5 eV and then to those with ≤1.0 eV (in this last case the number of systems being around 1490), the predictive accuracy improved markedly: test RMSE dropped from ∼0.34 to ∼0.19–0.21, with MAE following the same trend, indicating that the models capture the structure–property relationship much more reliably for narrower ranges for the barrier energy. Importantly, the train–test gap contracted steadily, from 0.25–0.32 to nearly 0.13–0.17. Further, at a very restrictive regime (<0.5 eV), roughly representing 500 2D materials, errors reached exceptionally low values (test RMSE 0.09–0.11, MAE 0.07), while the overfitting gap decreased to 0.07–0.10.

Regarding the ML results for the different atoms and for the aforementioned smaller energy windows, Se continuously reduced the absolute error and showed the largest decrease in RMSE and MAE. In contrast, Te emerged as the most robust and generalizable element, achieving the highest explained variance (*R* ^2^) and the smallest overfitting gap as the target range was narrowed. This suggests that for Te, the model captures a broader portion of the underlying variance without holding up spurious details. Finally, S remained a stable baseline with competitive performance and slightly smaller gaps in some intermediate regimes, but it was generally surpassed by Se in raw error and by Te in the variance explanation.

As a last technical remark, we observe that across all energy thresholds for all atoms, the hyperparameter search consistently selected shallow to moderately deep trees (depth ∼5–6), small learning rates (∼0.02), and moderate subsampling, while increasing regularization and reducing the effective number of boosting rounds as the barrier window narrowed—a coherent adjustment that limits complexity when overfitting risk decreases.

### 3.5 Interpretative Analyses

Beyond the standard fitting predictions enabled by ML models, one should not underestimate the importance of their potential interpretability so that the emerging patterns could, e.g., qualitatively unveil chemical traits. Ideally, a model should provide more than a numerical relationship extrapolating variations in the target variable as a function of the descriptors. It should also possess explanatory power, allowing one to attribute concrete physical and hierarchical significance to the observed correlations.

Hence, we apply to our results an interpretability protocol based on the usual implementation of the SHAP method. Briefly, rooted in Shapley cooperative game theory, SHAP transforms complex outputs into additive feature contributions, allowing for both local and global interpretations of nonlinear and otherwise opaque ML models (details of its usage and execution can be found in ref 35).

Next, we only consider XGBoost, as it exhibited the best overall performance. Moreover, we restrict the analysis to the better-performing energy regime (≤1.0 eV), comprising a set of 1495 2D structures. For each chalcogen (S, Se, and Te), the results are presented in Figure 6. The graphs depict the relative contributions of the 20 most relevant predictors—used in the XGBoost model to estimate the energy barriers—ranked in decreasing order of importance, from top to bottom. Each point specifies a given material. The horizontal axes give the SHAP index value, gauging the feature influence trend on the prediction. A positive (negative) index value indicates that the feature tends to increase (decrease) the barrier energy. A predictor has a higher (lower) value if its color is pinker (bluer).

**Figure 6:**
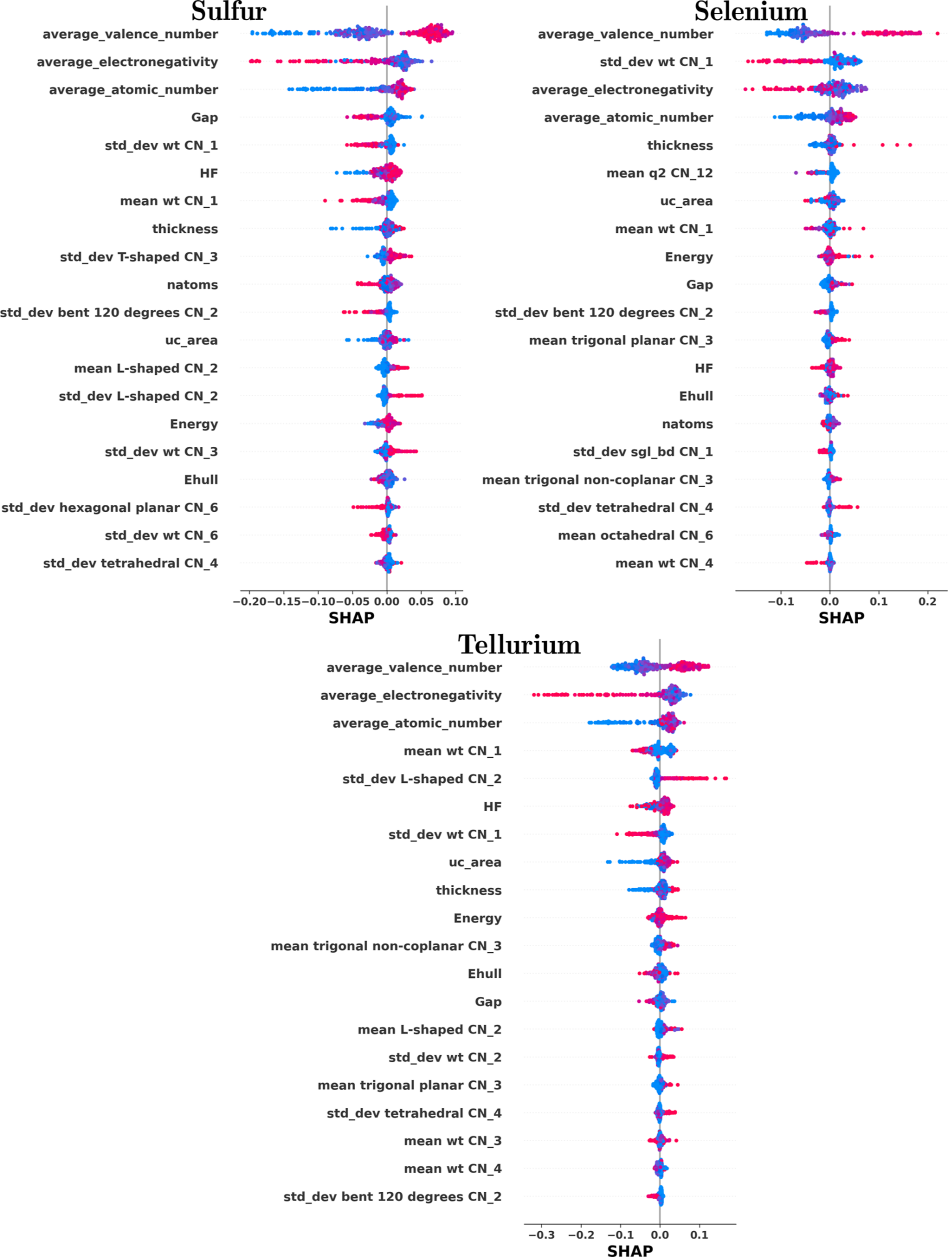
Interpretability analysis using the SHAP protocol applied to the energy barrier estimations from the XGBoost model. The graphs depict the results of adsorption of either S (top left), Se (top right), or Te (bottom) on the two-dimensional systems from the C2DB database with barrier energy ≤1.0 eV. Each point represents a distinct 2D material. Predictor features are ranked vertically in descending order of their importance in the model. A predictor variable value is high (low) if its color is pink (blue). The horizontal axis corresponds to the SHAP index. Positive (negative) values tend to increase (decrease) the energy barrier.

The relative importance of the top eight descriptors for each chalcogen atom is displayed in the left panels of Figure 7—in units of mean modulus SHAP value, horizontal axis. For the other 124 descriptors not shown, their combined importance in such units is 0.32 (S), 0.37 (Se), and 0.36 (Te). Below, we mostly discuss and interpret the top seven. Hence, the difference between the seventh and eighth SHAP index modulus gives an idea of the (somehow arbitrary) cutoff employed.

**Figure 7:**
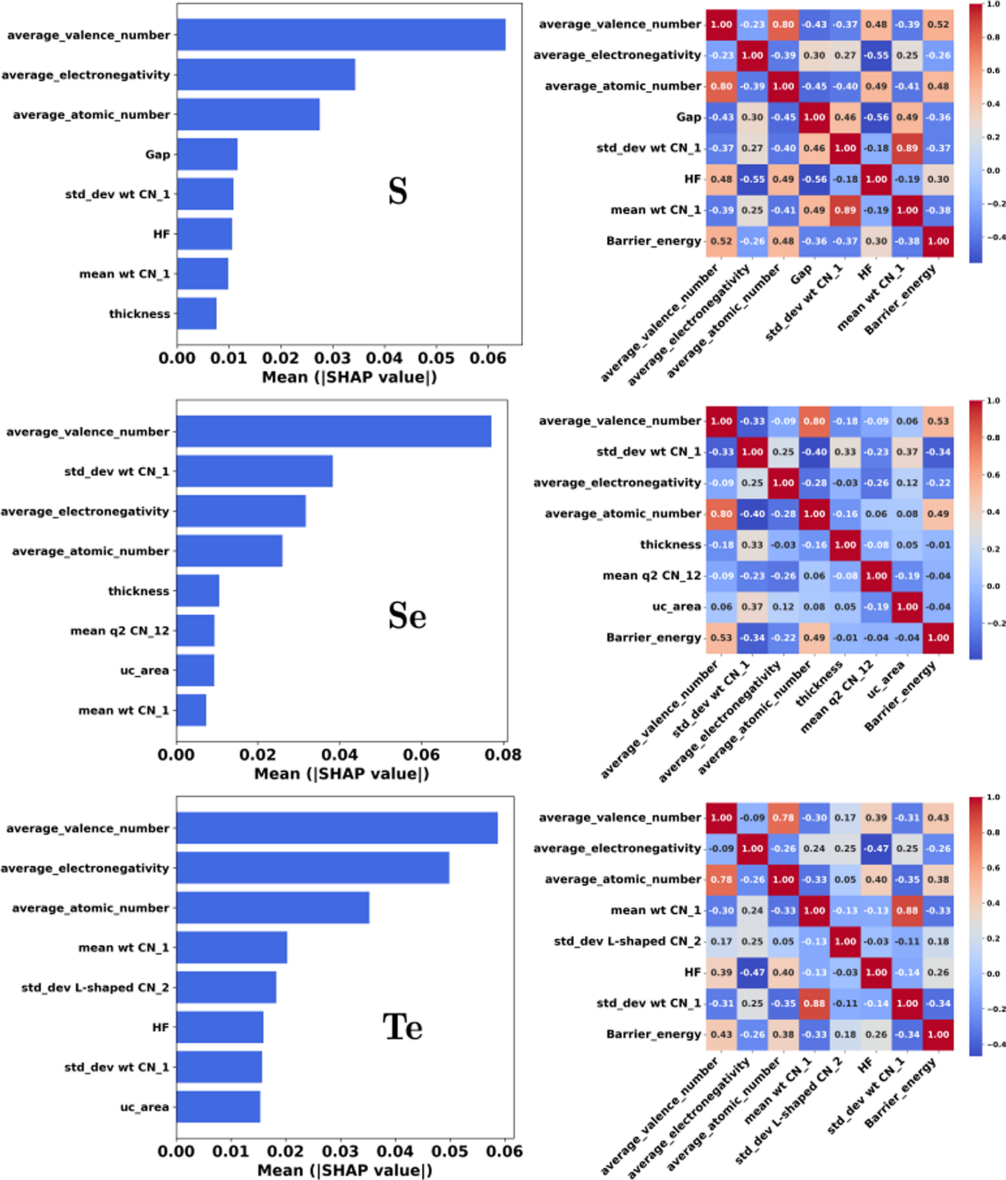
Top eight features from the SHAP analysis (left panels) and the classical Pearson correlation coefficients from the original FSD (right panels), for the 2D materials with S, Se, and Te atom impurity. The bar plots rank the most influential descriptors according to their mean absolute SHAP value. The heatmap matrices show the Pearson correlation coefficients between the seven most important features (as identified by SHAP for each atom) and the target barrier energy, computed directly from the FSD without any ML processing.

The SHAP results show that for the S atom, the top seven main influential features are

- average_valence_number
- average_electronegativity
- average_atomic_number
- Gap (Band Gap)
- std_dev wt CN_1 (Weighted Standard Deviation for Coordination Number 1 Atoms)
- HF (Heat of Formation)
- mean wt CN_1 (Weighted Standard Mean of Coordination Number 1 Atoms)

The plots in Figure 6 reveal that systems with a lower average valence number and atomic number and higher electronegativity (observe the color code) are more likely to exhibit lower barriers—negative SHAP values. This clearly aligns with S having higher chemical affinity for more electronegative and compact surfaces. Descriptors associated with surfaces with coordination number one also play a notable role, suggesting (typically) an easier diffusion of S in such structures. Although not among the top seven (see the above list and Figure 6), local topological features, like std_dev T-shaped CN_3 (ninth) and std_dev bent 120 °CN_2 (11th), are fairly relevant contributors, underscoring the influence of surface geometry in modulating the energy barrier.

For Se, the seven most relevant descriptors are

- average_valence_number
- std_dev wt CN_1
- average_electronegativity
- average_atomic_number
- thickness
- mean q2 CN_12 (mean of the order 2 Steinhardt bond orientation parameter for coordination number 12 atoms)
- uc_area (unit cell area)

Among the eight most important descriptors for Se, six overlap with those for S, although not in the same ranking order. However, Se displays a much higher sensitivity to the local orientation order variable mean q2 CN_12 and the average valence number feature; such latter feature’s SHAP values can be compared in Figure 7, which is around 22% higher for Se. This points to a more substantial role of surface disorder and irregularities, which is consistent with Se’s lower electronegativity, resulting in less localized interactions and, thus, a greater dependence on extended regions of the surface environment. Additionally, the thickness and unit cell area are also important descriptors. As seen in Figure 6, as thickness decreases and uc_area increases (although with a certain variability), the energy barrier decreases. This reinforces the relationship between the morphology and impurity mobility for Se.

For Te, the seven dominant features are

- average_valence_number
- average_electronegativity
- average_atomic_number
- mean wt CN_1
- std_dev L-shaped CN_2 (standard deviation for l-shaped coordination number 2 atoms)
- HF
- std_dev wt CN_1

Similar to Se and S, six of the eight most important descriptors for Te and S are identical. Moreover, four of these descriptors also coincide in their relative ranking of importance. This supports the fact that the distributions for S and Te in Figure 4 are fairly similar. The Te behavior is predominantly governed by average electronic properties, namely, valence, electronegativity, atomic number, and even HF (sixth descriptor), with a less pronounced influence from coordination-specific features; refer to Figure 7. These traits concur with Te’s chemical characteristics as a heavier, more polarizable, and less electronegative element.

More generally, Figure 6 reveals consistent trends for all three impurities while highlighting distinctive tendencies. On the one hand, the key electronic descriptors, i.e., average valence, atomic numbers, and electronegativity, are always among the top four features of S, Se, and Te. Therefore, they form the model core foundation given their strong discriminative power. On the other hand, S and Se show a balanced sensitivity to surface geometry and topology (as coordination type, layer thickness, and local disorder), whereas Te is primarily driven by average electronic properties, with a weaker influence from local structural variations, suggesting a more global and less directional interaction with the 2D surfaces. Also, the moderate relevance of features such as heat of formation (but less important for Se) indicates that thermodynamic stability and temperature-dependent properties should play a certain role in governing impurity diffusion in 2D structures, which is particularly relevant for applications in catalysis, sensing, and 2D material-based devices.

### 3.6 Pearson Correlation of the Main Descriptors

As an additional, independent assessment of the SHAP-derived hierarchy of descriptors—further evaluating their predictive power for barrier energy—we computed the corresponding Pearson correlation (see, e.g., ref 67) using the “raw” FSD, i.e., before any ML training.

The correlation matrices for the energy barrier and selected descriptors, the collection of seven features listed above for each chalcogen atom, are depicted in the right panels of Figure 7 (the energy barrier corresponds to the last row, or equivalently, last column). The color scale indicates both the sign and the strength of the relationship. Obviously, a given pair of descriptors does not need to have the same correlation value across the distinct chalcogen atom cases because of the way the correlations are computed^67^ and the collection of variables used.

Comparison between the two types of characterization (SHAP and Pearson) reveals that the simple calculation of linear statistical correlations, aiming to infer the most relevant quantities for a target property, is a circumscribed procedure. Indeed, the magnitude of a feature impact on the barrier energy as gauged by the “bare” correlation value is not always the same as indicated by SHAP, a much more sophisticated and complete nonlinear interpretability scheme. Notice that since the Pearson matrices have been constructed following the same ordering of predictors by SHAP (left panels), this fact is easy to check by inspecting the bottom row of each matrix. Differently from the SHAP analysis, along it, the associated coefficients |*c*_*ij*_| (taken in modulus to comply with the SHAP coefficient, also taken in modulus) do not monotonically decrease (from left to right).

Considering S, *i* being the Barrier_energy and *j* being (in decreasing order of importance, according to SHAP) average_valence_number, average_electronegativity, average_atomic_number, Gap, std_dev wt CN_1, HF, and mean wt CN_1, we have, respectively, |*c*_*ij*_| equal to 0.52, 0.26, 0.48, 0.36, 0.37, 0.30, and 0.38. Note that average_electronegativity (mean wt CN_1), the second (seventh) most important descriptor, has the lowest (third highest) Pearson correlation, 0.26 (0.38). So, based just on the raw data, one would conclude that mean wt CN_1 is more important than average_electronegativity to establish the barrier energy. Nonetheless, in full agreement with SHAP, investigations in the literature point to the opposite.^68–70^ Furthermore, ordering discrepancy is likewise observed for Se and Te. In particular, even if we restrict to the three top features according to SHAP, they are also classified as the three top by the Pearson correlation only for Se.

The matrices in the right panels of Figure 7 also show that the correlations between the seven most important predictors have a broad dispersion. While some are well-correlated, others are not. For the case of S, the top five features exhibit a significant degree of mutual correlation, with the smallest value of |*c*_*i*≠*j*_| equal to 0.23. Nonetheless, again considering the top five (even the top four), the variability is much stronger for Se and Te, with the lowest |*c*_*i*≠*j*_|’s being 0.03 and 0.09 for the former and 0.05 and 0.09 for the latter. These findings may suggest that, when considering the strength of their reciprocal correlations, there is no clear pattern for selecting the more pertinent predictors.

However, the above observations do not totally preclude the use of pure statistical measures, such as Pearson’s, to help identify characteristics of appropriate predictors for a model. As an illustration, from the last row of the matrices in Figure 7, we can calculate the standard deviation σ of $\left|c_{Barrier\text{\_}energyj}\right|$ , with *j* representing the seven most relevant features for S, Se, and Te. We find σ_Se_ = 0.220 > σ_S_ = 0.092 > σ_Te_ = 0.084. Although not very different for S and Te, these σ′s moderately point to a trend: the relative importance of the *j*’s is more evenly (unevenly) distributed in the case of Te (Se), with S in the middle way. This qualitatively agrees with the SHAP bar plots in Figure 7 and at least partially explains the distribution of barrier energies in Figure 4, which is wider for Se, narrower for S, and even narrower for Te. In fact, given the great diversity of 2D materials analyzed, a higher (lower) variability for the influence of the top predictors should lead to a bigger (smaller) dispersion of the resulting energy barrier, as seen in Figure 4.

### 3.7 Classifying the Materials: A Cluster Approach for S

The key premise in ML models is that suitably chosen descriptors can adequately estimate a target property, here the barrier energy. Accordingly, depending on the composition and structural characteristics of distinct material groups—determining the values of these predictors—the barrier energy is expected to fall within specific ranges. Conversely, the energy values could reveal systematic differences in the feature distributions, providing insight into structure–property classification of the systems, useful in applications.

To explore such a possibility, we employ the K-means clustering algorithm to divide the materials in the database according to their impurity mobility driving variables. Similar to the Pearson correlations, for independent analyses, we just address the FSD raw data, i.e., without any ML training. For simplicity, we discuss only S and the 2D systems for which the barrier energy is not superior to 1.0 eV (corresponding to 1495 materials). We leave Se and Te to the Supporting Information.

K-means is an iterative unsupervised protocol that partitions a data set into collections displaying similar attributes. This is done by calculating either the mean or median of the data-specified properties (in our present case, those in FSD). From these quantities, the scheme determines the clusters’ centroids so that distinct points can be arranged together into different sets. The protocol tries to minimize (maximize) the variance within each cluster (across clusters), so that data points in the same (different) cluster(s) are as similar (dissimilar) as possible. For a complete account of the procedure and implementation details, see, e.g., ref 71.

The results are summarized in Figure 8. The method has separated the full collection of 1495 2D materials into six (the optimal number found) distinct clusters, tagged from 0 to 5. The mean values of the top seven features for S (as classified by SHAP, see top left panel of Figure 7) as well as of the barrier energy in each cluster are presented as a matrix in Figure 8. The mean barrier energy increased with the cluster label. In the 3D plot of Figure 8, each bullet represents a specific material, also indicating the associated values of the top three descriptors. While the bullet colors identify which cluster they belong to, the diameters match the materials’ barrier energy values. The bar graph of Figure 8 depicts the distribution of barrier energies in each cluster.

**Figure 8:**
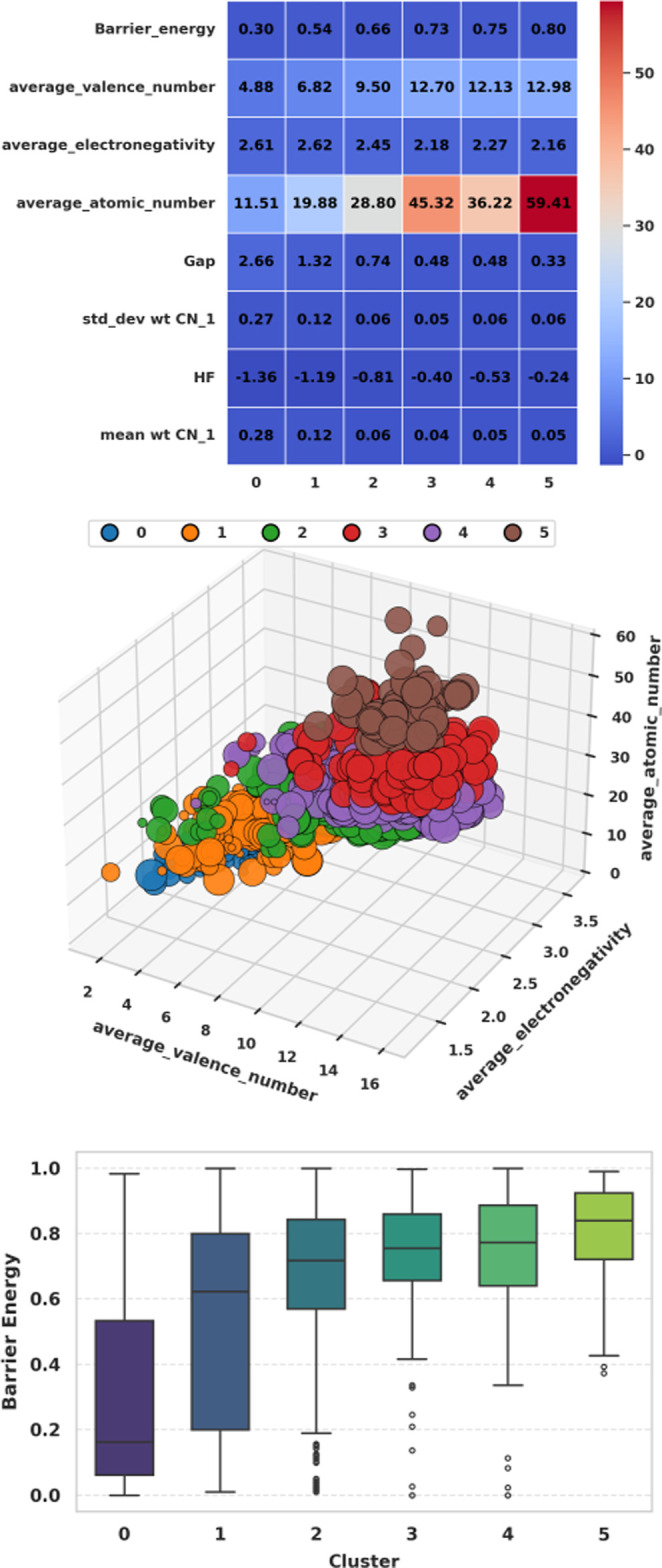
For S, materials of Figure 7 are classified as cluster 0 to 5 via *K*-means. Top: mean values of barrier energy and of seven relevant predictors (from SHAP) in each cluster. Middle: The materials clustering with the corresponding values of the three top predictors. A bullet size indicates the barrier energy value. Bottom: the barrier energy distribution in each cluster.

Among the clusters, distinct feature patterns are discernible. For example, the barrier energy average value (the first row of the matrix in Figure 8) increases through big jumps from clusters 0 to 1 (80.0%) and 1 to 2 (22.2%) and then with smaller variations from 2 to 3 (10.1%), 3 to 4 (2.7%) and 4 to 5 (6.7%)—refer also to the bar chart in Figure 8. However, this consistently relates to the distribution of barrier energy between materials within a cluster. Indeed, 1 and 0 (in this order) exhibit the greatest variations, with 2, 3, 4, and 5 showing reasonably similar narrower spread.

As for the average values of the seven primary features (matrix in Figure 8), the first, third, and sixth, namely, average_valence_number, average_atomic_number, and HF, increase with the cluster average barrier energy, the exception being cluster 4. The fourth descriptor, Gap, systematically decreases, again except for cluster 4. For the first and third descriptors, this can also be verified from the overall trend in the 3D plot of Figure 8. In particular, notice the broad distribution of cluster 4 in the average_valence_number axis and that typically the materials of cluster 3 are above those for cluster 4 along the average_atomic_number axis. We recall that these four predictors are highly correlated, as indicated by the Pearson matrix for S in Figure 7. The average_electronegativity (the second most important descriptor) exhibits a somewhat oscillatory trend: it remains nearly constant for clusters 0 and 1, decreases successively for clusters 2 and 3, and then shows a slight increase followed by a decrease for clusters 4 and 5, with a maximum difference of just 21% across all clusters. The fact that clusters 0 and 1 present the highest values of electronegativity is difficult to observe from the 3D plot in Figure 8 but becomes clear from Figure 9. The remaining two main predictors (fifth and seventh), associated with NC_1, exhibit a decaying trend toward a steady value as the mean barrier energy of the clusters increases.

**Figure 9:**
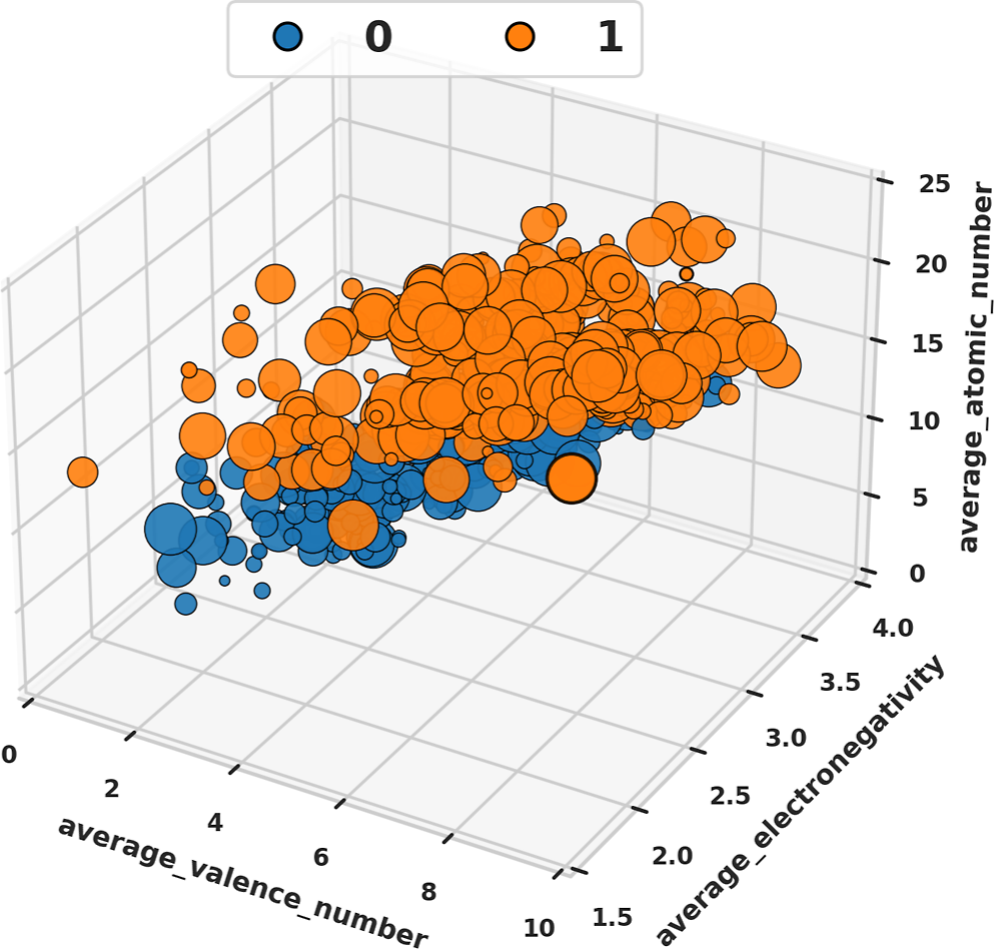
Same as in Figure 8, but showing only clusters 0 and 1. The plot highlights that materials in these clusters exhibit a broad distribution of electronegativity.

Lastly, we draw a comparison between *K*-means and SHAP, emphasizing that they are independent analyses that are not reliant on one another (except for selecting which descriptors to utilize in the former). For this, we recall that in Figure 6, the pink (blue) color indicates higher (lower) values for the predictors and that positive (negative) values of the SHAP index point to an increase (decrease) in the energy barrier. Thus, from Figure 6 for S, we clearly see that, in agreement with the *K*-means, the energy should increase with the first, second, and sixth predictors and decrease with the fourth one. The features associated with NC_1 tend to decrease the barrier only when they have high values; if they are small, their influence is less relevant (note in Figure 6 that the blue-coded materials have positive but close to zero SHAP index values). This is in accordance with the cluster characterization for these two descriptors. Finally, as predicted from the *K*-means, the somewhat more complex relationship between average_electronegativity and the barrier energy arises from the fact that materials with high values for such a predictor can either raise or lower the barrier (see the pink code-color on both sides of the SHAP axis in Figure 6 for this feature), while the majority of materials with lower values (blue code-color) tend to raise the barrier, albeit mildly. Hence, the SHIP and *K*-means are once again in line.

As a final remark, we observe that clustering approaches such as the simple and widespread *K*-means (but for more sophisticated ones see, e.g., ref 72) seem to point in the same direction as interpretative protocols, like SHAP. Nonetheless, the advantage of combining ML with interpretive models is to be able to directly determine the descriptor relevance order (refer to Figures 6 and 7), something not possible by just separating the materials into cluster classes.

## 4 Discussion and Conclusion

Over the past two decades, materials informatics has emerged as a transformative field, combining artificial intelligence and data science to explore distinct classes of complex systems. It focuses primarily on processing high-throughput data from large repositories to discover, screen, and characterize novel materials. With the advent of extensive chemical databases, such as the C2DB, the integration of ML models, robust statistical methods, novel optimization procedures, and evolutionary algorithms has enabled efficient inverse molecular design and automated materials analysis protocols.^73–75^ Along these lines, various AI strategies have been successfully deployed for structure forecasting and nanoscale distance estimation, aiming to accelerate computationally demanding ab initio methods through seamless hybrid workflows. For instance, crystal structure prediction frameworks such as USPEX,^76^ CALYPSO,^77^ and AIRSS^78^ have been widely adopted. Furthermore, ML platforms are increasingly utilized for both the classification and regression of interatomic distances—conceptually aligned with the philosophy outlined in Section 2.1.1 —such as mapping C–O bonds in lactone derivatives to correlate them with ring-opening reaction rates.^79^

In the present contribution, we have fully developed a novel framework to study a fundamental aspect of atomic diffusion in 2D systems: the migration energy barriers. To achieve this, we departed from conventional approaches in the literature; rather than merely speeding up expensive first-principles calculations, we sought to enhance semiempirical quantum methods—specifically the EHM—via the phenomenological determination of position-independent effective equilibrium distances (refer to eq 2). Moreover, by coupling these optimized semiempirical inputs with supervised ML, we significantly accelerated and improved the estimation of adsorption energy barriers. Crucially, we incorporated an interpretability protocol to extract concrete physical and chemical insights, thereby ascribing qualitative meaning to the typically opaque, black-box predictions of conventional ML architectures.

We emphasize that, in contrast to heavily scrutinized metal atom systems such as the aforementioned alkali metal adsorbates,^28–30^ our work focuses on the chalcogen S, Se, and Te impurities on 2D monolayers—a domain where detailed systematic surveys are conspicuously lacking. To the best of our knowledge, this work represents the most comprehensive and thorough investigation of chalcogen diffusion barriers presented in the literature.

Employing the EHM modified with a global mean equilibrium distance (*d*_eq_), we mapped the complete energy profiles of S, Se, and Te atoms adsorbed across 4036 distinct hosts from the C2DB library. From these profiles, average diffusion barriers were extracted to serve as target variables for our predictive ML pipelines. A diverse ensemble of physicochemical descriptors was generated via the Matminer library, supplemented by the custom feature engineering of averaged atomic properties. While several ML algorithms were systematically tested, the XGBoost regressor consistently outperformed alternative models in both statistical accuracy and generalization capacity. For the core ML investigations, the original materials library was narrowed down to structures exhibiting migration barriers under 2.0 eV (yielding approximately 2560 systems), as lower energy regimes represent the primary focus for practical nanoscale device applications.^16,46,69^

We verified that further restricting the target data set to low-barrier systems with energies below 1.0 eV (comprising 1495 structures) significantly enhances the overall fitting quality and predictive power. This behavior stems from the fact that transitioning from a broad 2.0 eV window to a highly concentrated subelectronvolt region stabilizes the underlying feature hierarchy. This reduction in data variance effectively mitigates overfitting, yields superior generalization, and establishes a dependable, physically intelligible link between macroscale migration barriers and atomic-scale descriptors.

To evaluate the present classification scheme performance, we confronted our predictions with DFT benchmarks using representative test sets for S and Se impurities, as shown in Tables 1 and 2. Tellurium was omitted to maintain a homogeneous level of theory across the benchmarks. As a heavy element (*Z* = 52), a rigorous description of Te requires the inclusion of effects like spin–orbit coupling^80^ and complex many–body interactions^81^ that scale drastically with reduced dimensionality. To avoid introducing structural and electronic asymmetries compared to the lighter S and Se chalcogens, a dedicated ab initio analysis for Te is deferred to a future study.

**Table 1:** Barrier Energies and Ranking Sequence for Sulfur (S) Adsorption^a^

material	DFT (eV)	material	EHM (eV)	EHM (×0.4) (eV)
Nb_3_OBr_7_	0.33	Na_2_Co_2_N_2_	0.20	0.08
Na_2_Co_2_N_2_	0.35	Re_6_Br_2_S_8_	0.50	0.20
Ba_4_Sb_4_F_4_S_8_	0.45	Ba_4_Sb_4_F_4_S_8_	1.50	0.60
Re_6_Br_2_S_8_	0.60	Ir_2_S_2_I_2_	2.40	0.96
Co_2_Te_3_	2.10	Nb_3_OBr_7_	3.50	1.40
Ir_2_S_2_I_2_	13.00	Co_2_Te_3_	4.50	1.80

a First-principles DFT benchmarks (ordered by ascending energy) are compared with the semi-empirical EHM and a global scaling of ×0.4 (reordered by ascending EHM values) for a representative set of 2D host materials, showcasing the model’s energy-regime hierarchy and resolution as outlined in the text.

**Table 2:** Barrier Energies and Ranking Sequence for Selenium (Se) Adsorption^a^

material	DFT (eV)	material	EHM (eV)	EHM (×0.5) (eV)
In_4_S_4_Br_4_	0.04	Re_4_S_8_	1.00	0.50
Re_4_S_8_	0.05	As_4_S_6_	1.50	0.75
Ta_2_CCl_2_	0.10	In_4_S_4_Br_4_	2.50	1.25
As_4_S_6_	0.125	Pd_2_I_2_Br_2_	3.50	1.75
Pd_2_I_2_Br_2_	2.67	Ta_2_CCl_2_	4.50	2.25
Nb_3_Pt_3_Te_14_	9.70	Nb_3_Pt_3_Te_14_	5.50	2.75

a First-principles DFT benchmarks (ordered by ascending energy) are compared with the semi-empirical EHM and a global scaling of × 0.5 (reordered by ascending EHM values) for a representative set of 2D host materials, showcasing the model’s energy-regime hierarchy and resolution as outlined in the text.

The six materials in Tables 1 and 2 were selected to span distinct electronic regimes, combining closely spaced, low-energy barriers to test model resolution with intermediate and high-energy values to evaluate hierarchy preservation. We report both DFT benchmarks and EHM values, introducing a scaling factor for conversion since quantitative coincidence is not expected; our framework prioritizes initial hierarchical screening over exact energy calculations, which are deferred to a subsequent fine-tuning stage. We remark that while a uniform scaling factor highlights systematic discrepancies, it represents a global optimization for this specific data set. A more sophisticated approach, potentially guided by ML, would employ separate, hierarchy-dependent factors to independently refine the low-, intermediate-, and high-energy regimes. Indeed, utilizing targeted parameters or ML models tailored to specific energy intervals or material families is a well-established strategy for enhancing semiempirical accuracy, as demonstrated in ref 82 for low-dimensional structural screenings.

As a consequence of the dense energy degeneracy observed at the top of both data sets, certain rank shuffles naturally occur within the low-energy regimes. For the sulfur set in Table 1, the first four compounds (Nb_3_OBr_7_, Na_2_Co_2_N_2_, Ba_4_Sb_4_F_4_S_8_, and Re_6_Br_2_S_8_) exhibit heavily compressed DFT values restricted to a narrow window between 0.33 and 0.60 eV. Similarly, for the selenium set in Table 2, the first four materials are tightly packed within an even smaller range of 0.04 to 0.125 eV. Predicting the exact sequential ordering within such subelectronvolt intervals exceeds the resolution limit of a simplified semiempirical framework. Nevertheless, the qualitative grouping is fundamentally correct, as the model successfully identifies and clusters these compounds as low-barrier materials, even preserving the exact intermediate rank of Ba_4_Sb_4_F_4_S_8_ (third position) in Table 1.

The intermediate and high-energy regimes, while preserving the overall ascending trend, expose specific pathological cases where semiempirical approximations deviate from ab initio physics. In Table 1, the model severely overestimates the barrier of Nb_3_OBr_7_ because the standard EHM method lacks charge-autoconsistency, which prevents it from properly relaxing the strong localized ionic polarization induced by the highly electronegative oxygen atom. Conversely, the massive underestimation of the Ir_2_S_2_I_2_ barrier (2.4 eV in EHM vs 13.0 eV in DFT) highlights a known limitation when treating heavy 5*d* transition metals, where strong electron correlation and spin–orbit coupling—both absent in EHM—play a dominant role in the electronic description. Similar electronic effects explain the quantitative underestimation of Nb_3_Pt_3_Te_14_ in Table 2 (5.5 eV in EHM vs 9.7 eV in DFT), where the fixed basis-set transferability of the semiempirical model faces limitations in capturing the intricate d-orbital hybridization between Niobium and Platinum under structural strain. However, the qualitative macro-trend remains resilient at the top of the scale, as shown by the fact that Nb_3_Pt_3_Te_14_ is unambiguously flagged by both methods as the highest diffusion barrier in Table 2, proving that the collective trade-off adjustments (factors of 0.4 and 0.5) successfully maintain a reliable physical hierarchy for presorting candidate materials.

The SHAP (SHapley Additive exPlanations) methodology was implemented to deconvolute the specific impact of each structural and electronic feature on the final energy barriers within the application-relevant ≤1.0 eV regime. This interpretability analysis confirmed that the correlations captured by XGBoost are chemically sound, robust across different impurity species, and reflective of the underlying migration physics. As anticipated, fundamental properties such as average valence number, electronegativity, and atomic number (*Z*) consistently ranked among the top-tier features determining the barrier heights. Concurrently, nonlocal topological and geometric features, including layer thickness and unit cell area, played a major role. Regarding spatial morphology, the SHAP analysis demonstrated that, as a general macro-trend (see Figure 6), thinner and structurally denser 2D frameworks effectively lower the atomic migration barriers.

Taking sulfur as a primary case study, independent statistical correlation measures were employed to corroborate the physical relevance of the descriptors selected by SHAP. In particular, Pearson correlation matrices and *K*-means clustering validated the internal consistency of the results achieved by our hybrid ML interpretability scheme. Nonetheless, the SHAP framework consistently outperformed standard statistical inference methods by capturing nonlinear interactions between descriptors that linear models naturally miss.

Importantly, our framework successfully uncovered nuanced behavioral differences among the three chalcogen impurities. For instance, the weighted standard deviation at coordination number 1 (Figure 7) reveals that selenium is remarkably sensitive to local geometric disorder. This indicates that surface imperfections and prolonged structural symmetry breaking exert a more pronounced influence on Se migration, a finding consistent with its lower electronegativity and more diffuse valence orbitals compared to sulfur.

For tellurium, the SHAP landscape is noticeably broader: while average valence and electronegativity still dictate the primary trends, local topology descriptors (such as the standard deviation of L-shaped CN_2_ and the weighted standard deviation of CN_1_) gain prominent roles (Figure 7). Because the Te barrier energy does not rely excessively on a narrow subset of parameters (contrasting with the slightly more parameter-sensitive Se), high variance in just a few isolated features does not trigger massive fluctuations in the prediction output across different hosts. This explains both the plateau-like profile observed in Figure 4 for Se and the physical reality of Te as a heavier, highly polarizable species that remains inherently resilient to subtle surface perturbations.

To conclude, we highlight two final remarks. First, this study demonstrates that when combined with robust ML models, simplified semiempirical approaches provide a highly viable, computationally cost-effective pathway to screen adsorption and kinetic properties across vast, uncharted 2D material databases. Moreover, the integration of posthoc interpretability tools elevates ML beyond its standard role as a mere numerical accelerator, transforming it into an active tool for extracting physical phenomenology.

Second, the current framework should not be viewed as an absolute replacement for high-level electronic structure calculations, but rather as an ultrafast presorting filter for extensive material libraries. A secondary stage of analysis can subsequently deploy rigorous ab initio modeling exclusively on the most promising candidate structures preselected for specific target applications.^83^ Although our findings are broadly supported by established literature trends, they are fundamentally semiempirical; hence, certain numerical discrepancies or localized breakdowns in phenomenological trends may occur when compared to full DFT calculations. For instance, on specific 2D hosts, the adatom could become permanently “anchored” within deep localized potential wells rather than undergoing continuous diffusion—a phenomenon that uniform parameters cannot fully capture. Indeed, mapped potential energy surfaces would ideally require a position-dependent parametrization (*h*_eq_) rather than a uniform effective *d*_eq_. We are currently developing a phenomenological *h*_eq_ correction model based on topological distance-to-closest-frame (DCF) descriptors,^84^ which we hope to report in the near future.

In summary, the key advantages of this framework lie in its exceptional computational speed, broad versatility, and rigorous physical interpretability, rendering it a powerful new asset for the high-throughput exploration of atomic transport phenomena in 2D systems.

## Supplementary Material



## Data Availability

All data and computational code artifacts required to reproduce the results reported in this work are openly available in the public GitHub repository accompanying this study: https://github.com/tromer-unb/chalcogen-barriers-2D-materials/tree/main. It contains the complete descriptor data sets used for the ML analyses of S, Se, and Te adsorption barriers in 2D materials, including both the Matminer-derived feature sets and the data sets employed in the correlation analyses. Furthermore, the repository provides the Jupyter notebook Barrier.ipynb, which reproduces the full computational workflow developed in this study, including data processing, supervised learning model construction, hyperparameter optimization, statistical evaluation, SHAP-based interpretability analyses, and the generation of all principal results. In this way, no additional data beyond that in the repository is needed to reproduce the findings presented in this article.

## Notes

Specific technical details and implementation guidelines, more details on the ML procedure, including data set splitting, cross-validation, and the final hyperparameters used in the XGBoost model, and additional results for the Se and Te, similar to those in the main text for the S (PDF)
