# Supplementary material for: Chalcogen Impurity Barriers in 2D Systems via Semi-Empirical/Machine Learning Modeling: A Survey of 4000 Materials
Source: J Chem Theory Comput. 2026 Jul 20;22(15):7854–72. doi: 10.1021/acs.jctc.6c00350 (PMC13472046; doi:10.1021/acs.jctc.6c00350)
Supplement: Supplementary file 1 [file ct6c00350_si_001.pdf]

Supporting Information: Chalcogen Impurity Barriers in 2D Systems via Semi-Empirical/Machine Learning Modeling: A Survey over 4000 Materials

M. L. Pereira, Jr,[†,‡] M. G. E. da Luz,[¶] P. Cesana,[§] A. L. da Rosa,[‖] M. J. Piotrowski,[⊥] D. Guedes-Sobrinho,[#] T. A. S. Pereira,[@,△,∇] E. A. Moujaes,[††] A. C. Dias,[‡‡] and R. M. Tromer[*,¶¶]

†*University of Brasília, College of Technology, Department of Electrical Engineering, Brasília-DF, 70910-900, Brazil.*

‡*Rice University, Department of Materials Science and NanoEngineering, Houston-TX, 77005, United States*

¶*Departamento de Física & Multidisciplinary Laboratory for Modeling and Analysis of Data in Complex Systems (MADComplex), Núcleo de Modelagem e Computação Científica-Centro Interdisciplinar de Ciência, Tecnologia e Inovação, Universidade Federal do Paraná, 81531-980 Curitiba-PR, Brazil*

§*Institute of Mathematics for Industry, Kyushu University, 744 Motooka, Fukuoka 819-0395, Japan*

‖*Institute of Physics, Federal University of Goiás, Campus Samambaia, 74690-900, Goiânia, Goiás, Brazil.*

⊥*Department of Physics, Federal University of Pelotas, PO Box 354, Pelotas, RS,* 96010 − 900, *Brazil*

#*Chemistry Department, Federal University of Paraná, CEP* 81531 − 980, *Curitiba, Brazil*

@*Physics Graduate Program, Institute of Physics, Federal University of Mato Grosso, Cuiabá, MT, Brazil*

△*Instituto de Física, Universidade Federal Fluminense, 24210-346, Niterói, Rj, Brasil*

∇*National Institute of Science and Technology on Materials Informatics, Campinas, Brazil*

††*Institute of Physics, Department of Solid State Physics, Federal University of Bahia, Campus Ondina, 40170-115, Salvador, BA, Brazil.*

‡‡*Institute of Physics and International Center of Physics, University of Brasília, 70919-970, Brasília, DF, Brazil.*

¶¶*University of Brasília, Institute of Physics, Brasília-DF, 70910-900, Brazil*

E-mail: raphael.tromer@unb.br

This Supplementary Information (SI) provides a systematic description of the machine learning (ML) models used to predict the average adsorption energy barriers of two-dimensional (2D) systems. While general methodological aspects regarding dataset, features used, model training, and validation are described in the main text, this document focuses more specific technical details and implementation guidelines. It also presents addition results for to the Selenium (Se) and Tellurium (Te), similar to those in the main text for the

Sulfur (S). Specifically, for Se and Te compare the predicted and actual barrier values for the 4036 2D materials selected from the Computational 2D Materials Database (C2DB).[S1] To quantify and compare model performance, we utilize three standard evaluation metrics: the coefficient of determination (R^2), mean absolute error (MAE), and mean squared error (MSE).

Details of the Semi-Empirical Computations

This work involved the construction and analysis of a comprehensive dataset comprising 4036 two-dimensional materials extracted from the C2DB database. For each structure, energy barriers for the adsorption of S, Se, and Te were computed using the semi-empirical Extended Hückel Method (EHM). These values served as target variables for subsequent machine learning modeling.

All adsorption energy barrier calculations were performed using the EHM as implemented in the YAeHMOP package. The default atomic orbital parameters provided by YAeHMOP were employed for all elements present in the dataset. These parameters include tabulated valence orbital energies and Slater-type orbital exponents, ensuring a consistent and transferable description across the diverse set of 2D materials extracted from the C2DB database.

The off-diagonal Hamiltonian matrix elements were constructed using the Wolfsberg–Helmholtz approximation:

$$H_{ij} = K\, S_{ij}\, \frac{(H_{ii} + H_{jj})}{2}, \qquad (1)$$

where H_{ij} and S_{ij} are the Hamiltonian and overlap matrix elements, respectively, and K is the empirical Wolfsberg–Helmholtz constant. In this work, a value of $K = 3.5$ was adopted. This value was selected after preliminary benchmarking tests on graphene, where it yielded adsorption-related energy trends in closer agreement with reference density functional theory (DFT) results compared to the standard default values.

All structures were obtained from the Computational 2D Materials Database (C2DB) and used without further structural relaxation within the EHM framework, since the goal of this work is large-scale screening rather than high-precision total energy optimization. For each material, adsorption configurations for S, Se, and Te impurities were evaluated using a consistent geometric protocol to ensure comparability across the 4036 systems.

The average adsorption energy barrier was computed from the EHM electronic structure by evaluating the energy difference between the adsorbed and reference configurations following the same procedure adopted throughout the dataset. This semi-empirical approach enables the efficient calculation of adsorption trends over thousands of heterogeneous 2D materials while maintaining physically meaningful chemical trends, as previously validated against DFT benchmarks for representative systems such as graphene.

Application of Machine Learning Models Across the 2D Material Dataset

Descriptor Extraction

Each material was characterized by a rich set of physicochemical descriptors, derived from C2DB and extended using the Matminer library. These descriptors capture a wide range of information, including:

- *Electronic properties*: average and maximum electronegativity, valence electron counts, Fermi energy, thickness, etc;
- *Structural and geometric features*: lattice parameters, coordination numbers, density, dimensionality, and symmetry information;
- *Atomic-level statistics*: elemental averages, weighted sums, and variances for atomic number, atomic radius, and polarizability.

The resulting feature vectors formed the input for supervised regression models.

Model Training and Evaluation

Four regression models were trained and evaluated using the prepared dataset: Linear Regression, Decision Tree Regressor, Neural Network (Multilayer Perceptron) and XGBoost Regressor. Hyperparameter optimization was performed using Optuna, and model performance was assessed via the R^2 score and mean absolute error (MAE) on the held-out test set. The XGBoost model consistently outperformed the others across all adsorbates, achieving the highest predictive accuracy.

Interpretability

To interpret the predictions, SHAP analysis was applied to the XGBoost model. This allowed identification of the most influential descriptors for each chalcogen species. The analysis highlighted the importance of electronic descriptors such as valence electron count and electronegativity, with geometric and topological features contributing more prominently in the case of S and Se, while Te showed a smoother profile influenced by global structural properties.

Machine Learning Implementation Details

All machine learning (ML) models were implemented in Python (version 3.10) using a standardized and reproducible workflow. The main libraries employed in this study were `scikit-learn` for classical regression models (Linear Regression, Decision Tree Regressor, and Multilayer Perceptron), `xgboost` for the gradient boosting model, `matminer` for descriptor handling and feature integration, and `shap` for model interpretability analysis. Additional data manipulation and numerical operations were performed using `pandas` and `numpy`.

To ensure full reproducibility of the results, fixed random seeds were used throughout all stages of the ML pipeline, including dataset splitting, model initialization, and hyperparameter optimization. Specifically, a global random seed of 42 was adopted for NumPy, scikit-learn, XGBoost, and Optuna whenever applicable.

The dataset was randomly divided into training (80%) and testing (20%) subsets using a seeded random shuffle to maintain consistency across all models and chalcogen species. Feature matrices and target vectors were processed using the same split to avoid data leakage.

All models were trained using the optimized hyperparameters obtained via Optuna-based Bayesian optimization. The training procedures were executed on a standard CPU environment, without GPU acceleration, ensuring methodological consistency and accessibility for large-scale screening applications.

Model evaluation metrics, including the coefficient of determination (R^2), mean absolute error (MAE), and mean squared error (MSE), were computed using the `scikit-learn` metrics module under identical conditions for all models to allow a fair and systematic comparison.

Data Preprocessing and Validation Strategy

Prior to training the machine learning models, the full set of descriptors (FSD) was subjected to a standardized preprocessing pipeline to ensure numerical stability, reproducibility, and fair model comparison across the heterogeneous collection of 2D materials from the C2DB database.

First, duplicate entries and non-physical data points were removed when present. Missing descriptor values generated during the Matminer featurization process were handled through consistent filtering to avoid bias introduced by imputation, given the large size of the dataset. All features were inspected for numerical consistency and outliers associated with clearly non-representative structures were excluded only when they corresponded to anomalous barrier energies outside the physically meaningful range considered in this work.

Since the descriptor space contains variables with different physical scales (e.g., electroneg-

ativity, atomic number, thickness, unit cell area), feature normalization was applied when required by the model, particularly for the Multilayer Perceptron (MLP), using standard scaling (zero mean and unit variance). Tree-based models (Decision Tree and XGBoost), which are intrinsically scale-invariant, were trained using the original descriptor magnitudes to preserve their physical interpretability.

The complete dataset, comprising 4036 two-dimensional materials (and 12,108 entries when considering the three chalcogen adsorbates), was divided into training (80%) and testing (20%) subsets using a stratified random shuffle with a fixed seed. This hold-out strategy was chosen due to the large size, compositional diversity, and structural heterogeneity of the C2DB database, which includes materials spanning multiple crystal families, electronic structures, and chemical compositions. Under such conditions, an 80/20 split provides a statistically representative sampling of the descriptor space while preserving a sufficiently large independent test set for robust generalization assessment.

To further verify the reliability of the adopted partitioning scheme, additional cross-validation tests (k-fold) were performed during the hyperparameter optimization stage, particularly for the XGBoost model. The consistency between cross-validation scores and the held-out test performance, as discussed in the main text, indicates that the models do not suffer from significant information leakage and that the predictive trends are not artifacts of a specific data split.

It is worth emphasizing that, in materials informatics applications involving large and highly heterogeneous databases such as C2DB, a carefully controlled hold-out validation is often preferred over exhaustive k-fold cross-validation. This is because the primary goal is large-scale screening and generalization across diverse chemical spaces, rather than local interpolation within a narrow compositional domain. Therefore, the adopted validation protocol ensures both computational efficiency and methodological robustness for high-throughput prediction of adsorption energy barriers.

Clustering Analysis (k-means)

To complement the supervised learning and interpretability analyses, an unsupervised clustering approach based on the k-means algorithm was employed to identify natural groupings of 2D materials according to their physicochemical descriptors and associated adsorption energy barriers.

The clustering was performed using the raw Full Set of Descriptors (FSD), without prior machine learning training, in order to provide an independent statistical characterization of the dataset. Only the most relevant descriptors identified through the SHAP analysis were used as input features for the clustering stage, ensuring physical interpretability and reducing redundancy in the high-dimensional descriptor space.

Before applying k-means, the selected features were standardized (zero mean and unit variance) to prevent descriptors with larger numerical magnitudes (e.g., unit cell area or atomic number) from dominating the Euclidean distance metric used by the algorithm. This normalization step is particularly important given the heterogeneous nature of the C2DB-derived descriptor set, which combines electronic, structural, and compositional variables.

The optimal number of clusters (k) was determined through an internal validation procedure based on the elbow method and variance minimization, balancing model simplicity and descriptive power. The final choice of $k = 6$ (for the S case discussed in the main text) provided well-separated clusters with physically meaningful trends in barrier energy and descriptor distributions.

The k-means algorithm was initialized with multiple random centroid seeds to ensure convergence toward stable partitions and to minimize sensitivity to initialization effects. Clustering results were analyzed in terms of: (i) mean descriptor values per cluster, (ii) barrier energy distributions, and (iii) multidimensional projections using the most influential predictors. This unsupervised classification enabled the identification of systematic structure–property relationships across compo-

sitionally and electronically distinct families of 2D materials, independently corroborating the trends revealed by the SHAP interpretability analysis.

ML, Interpretability Model and Clustering Analysis Parameters

For each chalcogen impurity, the corresponding descriptor dataset was processed independently. The target variable was the average adsorption energy barrier, denoted as `media` in the raw input files. Prior to model training, non-descriptor features — specifically `formula`, `ind`, and the directional barrier components `max_valorX`, `max_valorY`, and `max_valorZ` — were excluded from the datasets.

For the sulfur (S) dataset, four supervised regression models were evaluated. A standard linear regression model was first employed as a baseline without hyperparameter tuning. Second, a decision tree regressor was trained with a maximum depth of 6 and `random_state` = 42. Third, a multilayer perceptron (MLP) regressor was implemented with the following architecture and optimization constraints: two hidden layers containing 60 and 15 neurons, respectively, Rectified Linear Unit (ReLU) activation functions, the Adam optimizer, an L_2 regularization parameter $\alpha = 1.5 \times 10^{-4}$, a maximum of 1000 iterations, and `random_state` = 42.

The best-performing model across all scenarios was the XGBoost regressor. The optimized hyperparameters obtained for the S, Se, and Te datasets are summarized below in Eqs. (2), (3), and (4), respectively, for S, Se and Te:

$$\begin{aligned} &\texttt{n\_estimators} = 292,\\ &\texttt{learning\_rate} = 0.03441590896144655,\\ &\texttt{max\_depth} = 12,\\ &\texttt{subsample} = 0.6662215207801131,\\ &\texttt{colsample\_bytree} = 0.6003904138832606,\\ &\texttt{reg\_lambda} = 0.311000323561672,\\ &\texttt{random\_state} = 42. \end{aligned} \tag{2}$$

$$\begin{aligned} &\texttt{n\_estimators} = 337,\\ &\texttt{learning\_rate} = 0.052014211178989106,\\ &\texttt{max\_depth} = 11,\\ &\texttt{subsample} = 0.8411806864686558,\\ &\texttt{colsample\_bytree} = 0.9468709356630377,\\ &\texttt{reg\_lambda} = 5.925657514455248,\\ &\texttt{random\_state} = 42. \end{aligned} \tag{3}$$

$$\begin{aligned} &\texttt{n\_estimators} = 492,\\ &\texttt{learning\_rate} = 0.038921216034914774,\\ &\texttt{max\_depth} = 9,\\ &\texttt{subsample} = 0.5922533267068893,\\ &\texttt{colsample\_bytree} = 0.9353365959889494,\\ &\texttt{reg\_lambda} = 7.1907361927013485,\\ &\texttt{random\_state} = 42. \end{aligned} \tag{4}$$

Following the training phase, SHAP (SHapley Additive exPlanations) analyses were conducted on the held-out test sets to evaluate feature importance. Feature relevance was quantified via the mean absolute SHAP values, and the eight most dominant descriptors were selected for further visualization. Pearson correlation matrices were subsequently computed for these reduced descriptor subsets after standardizing the numerical features using a `StandardScaler` protocol. Finally, k-means clustering was applied to the selected descriptor space to identify structural patterns, configured with six clusters, `random_state` = 42, and `n_init` = 10 for the final initialization pass.

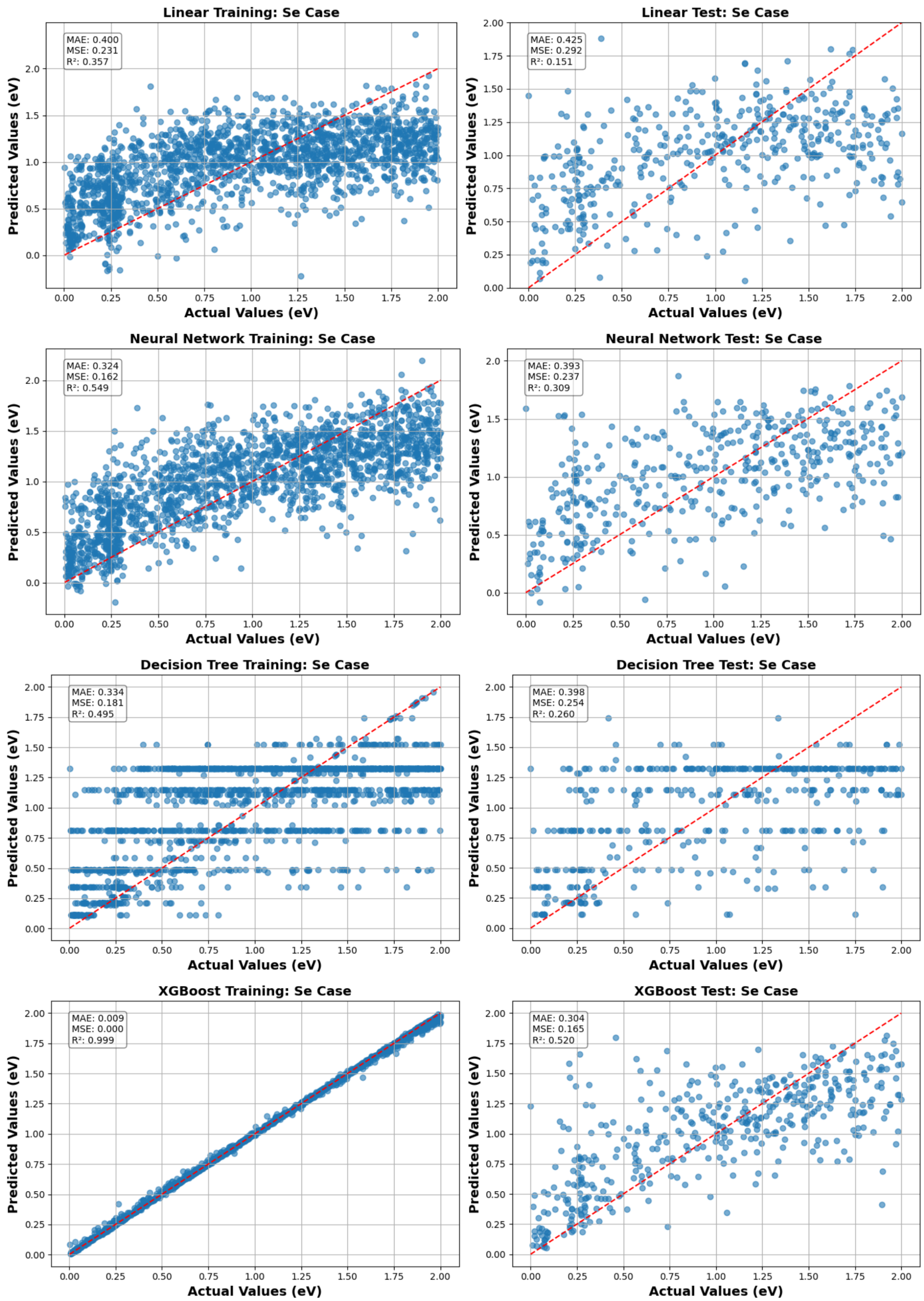

Figure S1: Scatter plots comparing predicted and actual values of the average adsorption energy barriers (in eV) for Selenium impurities on 4036 two-dimensional systems from the C2DB database, using four machine learning models: Linear Regression (first row), Neural Network (second row), Decision Tree (third row), and XGBoost (fourth row). Left plots show the training phase; right plots show the testing phase. The red dashed line indicates the ideal $y = x$ line. Performance metrics include MAE, MSE, and R^2.

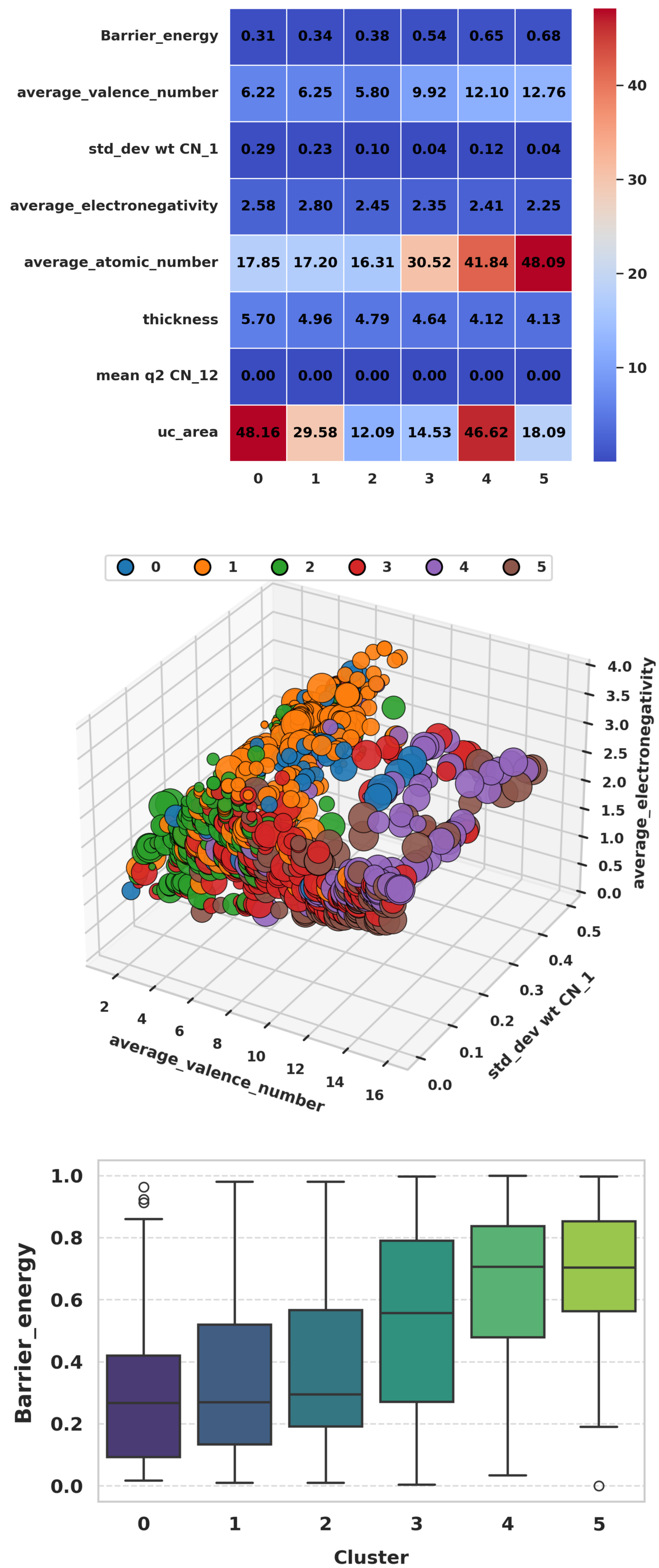

Figure S2: The heatmap (top) summarizes the mean values of key structural and electronic descriptors within each k-means cluster for Se, highlighting distinct physicochemical profiles. The 3D scatter plot (middle) visualizes the clustering landscape across $average\_valence_number$, std_dev wt_CN_1, and $average\_electronegativity$, with point size proportional to $Barrier\_energy$. The boxplot (bottom) compares the distribution of $Barrier\_energy$ across clusters, revealing systematic differences in energy barriers among compositionally and electronically distinct groups.

Results for Se and Te

Machine learning models, correlation analysis and cluster classification applied to the Se case

Figure S1 displays the performance of four ML models—Linear Regression, Neural Network (Multilayer Perceptron, MLP), Decision Tree, and XGBoost—applied to Se adsorption barrier prediction. The left panels show the training results, while the right panels correspond to the test phase.

In the first row, the Linear Regression model exhibits limited predictive capability. Although it reaches $R^2 = 0.357$ on the training set, the performance decreases significantly during testing ($R^2 = 0.151$, MAE = 0.425 eV), reflecting poor generalization. The widespread deviation from the identity line ($y = x$) indicates that the linear approximation fails to capture the nonlinear interactions between structural and chemical descriptors and the adsorption barriers. This result is consistent with the behavior observed for sulfur in the main text and reflects the limitations of linear models, particularly when dealing with complex descriptor landscapes.

The Neural Network model, shown in the second row, offers a clear improvement. With $R^2 = 0.549$ in training and $R^2 = 0.309$ in testing, the model reduces MAE and MSE values relative to Linear Regression. Despite these gains, a noticeable bias remains: the model underestimates high barrier values and overestimates low ones, as evidenced by the flattened slope relative to the $y = x$ reference. This effect likely stems from the limited expressiveness of the current architecture and could be mitigated by deeper networks or ensemble strategies. The results demonstrate that neural networks can accommodate nonlinearity and provide better generalization than simpler approaches.

The Decision Tree model (third row) yields mixed results. While it performs reasonably during training ($R^2 = 0.495$, MAE = 0.334 eV), its performance declines in testing ($R^2 = 0.260$, MAE = 0.398 eV), suggesting overfitting. The staircase-like pattern in the scatter plots highlights the model's tendency to produce discrete outputs, which is incompatible with the continuous nature of the target property. These issues are typical of single-tree approaches when applied to high-dimensional or noisy datasets and limit their applicability in precise regression tasks.

XGBoost, shown in the fourth row, consistently outperforms the other models. It nearly achieves a perfect fit with $R^2 = 0.999$ and an almost negligible MAE (0.009 eV) in training. The test performance remains robust ($R^2 = 0.520$, MAE = 0.304 eV, MSE = 0.165), indicating strong generalization. XGBoost leverages ensemble learning and internal regularization to balance bias and variance effectively. Its ability to model complex feature-target relationships and resist overfitting makes it suitable for large-scale screening tasks involving heterogeneous 2D systems.

Note that all models follow the same general trends observed for sulfur in the main manuscript. Linear Regression and decision trees lack the flexibility required to model the system accurately, while neural networks show moderate success. XGBoost is the most reliable and efficient approach for predicting selenium adsorption energy barriers in 2D materials.

Finally, clustering classification (via k-means) analysis, as done for S in the main text, is shown in Fig. S2 for Se.

Machine learning models, correlation analysis and cluster classification applied to the Te case

Figure S3 presents the ML model performances for Te impurities, using the same framework applied to the Se and S cases.

The tellurium case follows similar trends but benefits from the lower variance in energy barrier distribution. This characteristic slightly improves the performance of simpler models. Linear Regression achieves its highest test R^2 among all chalcogens ($R^2 = 0.319$), although it still fails to account for high-barrier outliers. The Neural Network model shows modest gains, with a smoother prediction profile and MAE re-

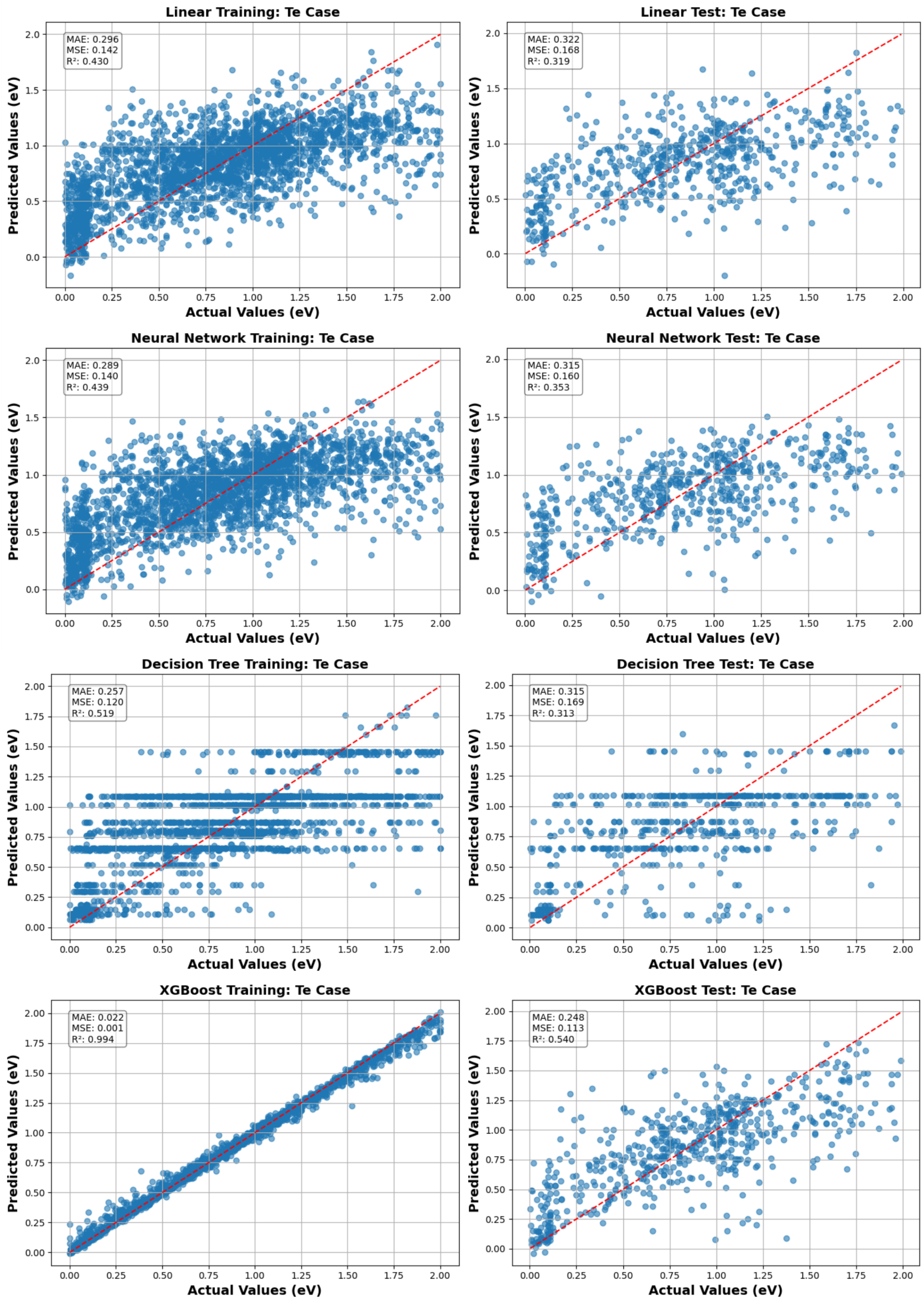

Figure S3: Scatter plots comparing predicted and actual average adsorption energy barrier values (in eV) for Tellurium impurities on 4036 two-dimensional systems from the C2DB database, using four machine learning models: Linear Regression (first row), Neural Network (second row), Decision Tree (third row), and XGBoost (fourth row). Left plots correspond to training and right plots to testing. The red dashed line represents the ideal $y = x$ reference. Performance metrics include MAE, MSE, and R^2.

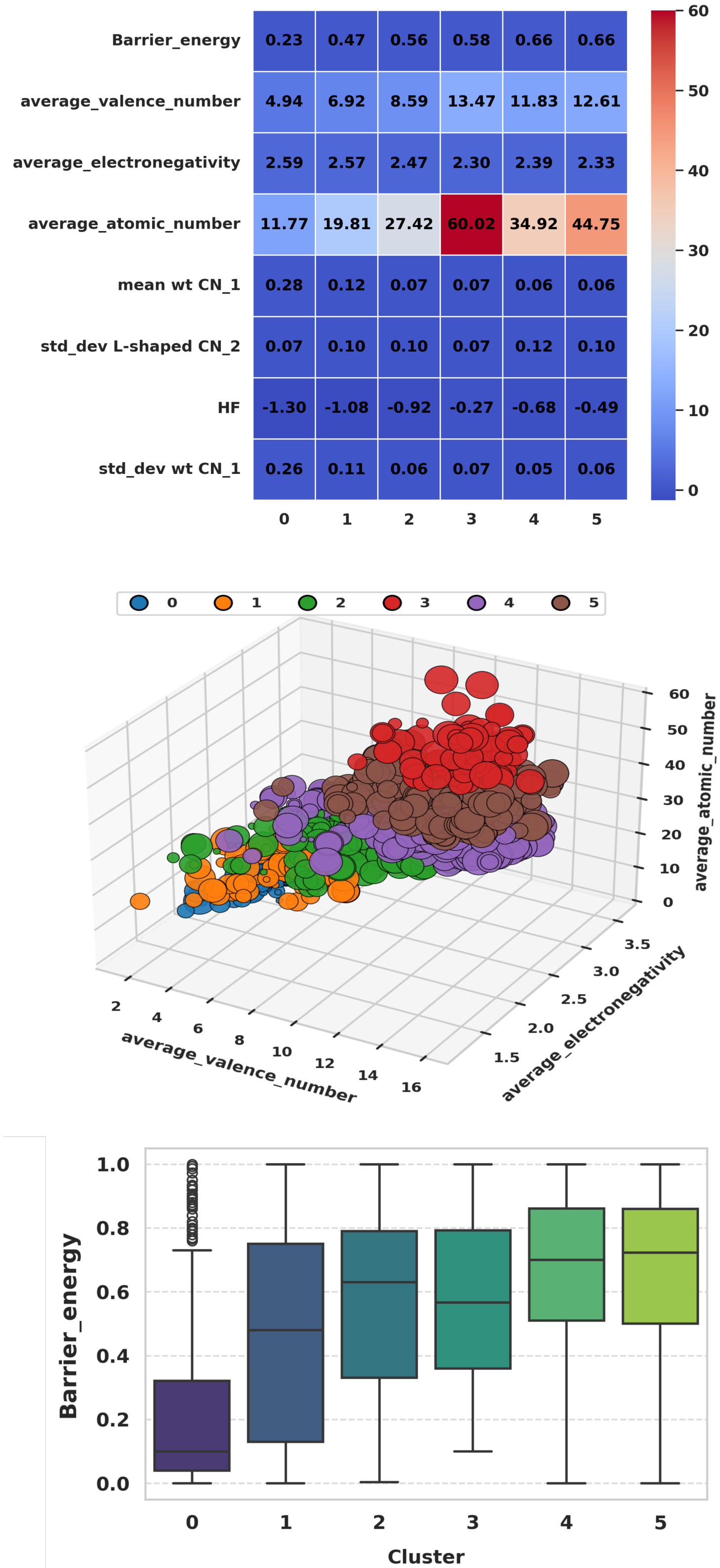

Figure S4: The heatmap (top) summarizes the mean values of key structural and electronic descriptors within each k-means cluster for Te, highlighting distinct physicochemical profiles. The 3D scatter plot (middle) visualizes the clustering landscape across $average\_valence_{n}umber$, std_dev wt_CN_1, and $average\_electronegativity$, with point size proportional to $Barrier\_energy$. The boxplot (bottom) compares the distribution of $Barrier\_energy$ across clusters, revealing systematic differences in energy barriers among compositionally and electronically distinct groups.

duced to 0.315 eV.

The Decision Tree model again overfits, achieving good training scores ($R^2 = 0.519$) but declining in testing ($R^2 = 0.313$), with discrete prediction artifacts remaining. XGBoost outperforms, reaching $R^2 = 0.540$ and MAE = 0.248 eV on the test set. Its ability to adapt across varying levels of complexity confirms its robustness.

Finally, clustering classification (via k-means) analysis, as done for S in the main text, is shown in Fig. S4 for Te.

Concluding Remarks About the Se and Te Results

The results presented for Se and Te reinforce the general trends discussed in the main text. While models like linear regression and decision trees may perform slightly better in cases with lower property variability, such as tellurium, they remain limited in capturing complex patterns. Neural networks offer moderate improvements, but XGBoost consistently delivers the best results across all chalcogen impurities analyzed. Its accuracy, robustness, and efficiency make it the most reliable approach for large-scale prediction of adsorption energy barriers in two-dimensional materials.

References

(S1) Gjerding, M. N.; Taghizadeh, A.; Rasmussen, A.; Ali, S.; Bertoldo, F.; Deilmann, T.; Knøsgaard, N. R.; Kruse, M.; Larsen, A. H.; Manti, S.; Pedersen, T. G.; Petralanda, U.; Skovhus, T.; Svendsen, M. K.; Mortensen, J. J.; Olsen, T.; Thygesen, K. S. Recent progress of the Computational 2D Materials Database (C2DB). *2D Materials* **2021**, *8*, 044002.
